# An epithelial *GPR35* isoform supports tumor‐associated transcriptional and metabolic phenotypes

**DOI:** 10.1002/1873-3468.70423

**Published:** 2026-08-01

**Authors:** Jørgen D. Rønneberg, Martine Hjeltnes, Josephine Krieger, Joshua E. Elias, Maria Stensland, Kristian Holm, Xiaojun Jiang, Chuki Khangsar, Tuula A. Nyman, Arthur Kaser, Johannes R. Hov, Tom H. Karlsen, Nicole C. Kaneider, Frode L. Jahnsen, Georg Schneditz

**Affiliations:** ^1^ Division of Surgery and Specialized Medicine, Norwegian PSC Research Center Oslo University Hospital Rikshospitalet Norway; ^2^ Division of Surgery and Specialized Medicine, Research Institute of Internal Medicine Oslo University Hospital Rikshospitalet Norway; ^3^ Faculty of Medicine, Institute of Clinical Medicine University of Oslo Norway; ^4^ Cambridge Institute of Therapeutic Immunology and Infectious Disease, Jeffrey Cheah Biomedical Centre University of Cambridge UK; ^5^ Division of Gastroenterology and Hepatology, Department of Medicine University of Cambridge, Addenbrooke's Hospital UK; ^6^ Department of Immunology University of Oslo and Oslo University Hospital Norway; ^7^ Proteomics Core Facility University of Oslo and Oslo University Hospital Norway; ^8^ Section of Gastroenterology, Department of Transplantation Medicine, Division of Surgery and Specialized Medicine Oslo University Hospital Rikshospitalet Norway; ^9^ Department of Pathology, Oslo University Hospital and Institute of Clinical Medicine University of Oslo Norway

**Keywords:** cell metabolism, cell signaling, colon cancer, G protein‐coupled receptor, molecular pharmacology

## Abstract

G protein‐coupled receptor 35 (GPR35) has been implicated in cancer, but the functional roles of its isoforms remain unresolved. Here, we characterize GPR35‐long, an N‐terminally extended epithelial isoform selectively enriched in colorectal cancer and cholangiocarcinoma. Mass spectrometry and immunohistochemistry confirmed GPR35‐long protein expression in tumor cells. Functional analyses revealed that GPR35‐long supports tumor‐associated transcriptional programs, enhances cellular ATP production, and exhibits increased constitutive and ligand‐induced beta‐arrestin signaling. These phenotypes were selectively suppressed by the inverse agonist CID‐2745687, identifying GPR35‐long as a functionally distinct isoform with selective pharmacological sensitivity in tumor cells.

## Graphical abstract

*GPR35* generates two functionally distinct isoforms with previously unresolved roles. *GPR35*‐short mediates immune‐cell chemotaxis, while *GPR35*‐long is enriched in colorectal cancer epithelium, where it supports increased metabolism, proliferation, and tumor‐associated transcriptional programs. Pharmacological blockade with the inverse agonist CID‐2745687 selectively suppresses these phenotypes, identifying GPR35‐long as a promising target in gastrointestinal cancer biology.

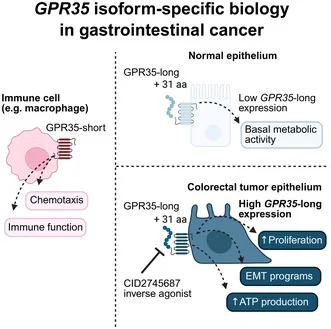

## Abbreviations

**ATP**, adenosine triphosphate

**CCA**, cholangiocarcinoma

**CRC**, colorectal cancer

**CRISPR**, clustered regularly interspaced short palindromic repeats

**DEG**, differentially expressed gene

**ECAR**, extracellular acidification rate

**ECM**, extracellular matrix

**EMT**, epithelial‐mesenchymal transition

**ERCP**, endoscopic retrograde cholangiopancreatography

**FCCP**, carbonyl cyanide‐p‐trifluoromethoxyphenylhydrazone

**GO**, gene ontology

**GPCR**, G protein‐coupled receptor

**GSEA**, gene set enrichment analysis

**IBD**, inflammatory bowel disease

**IHC**, immunohistochemistry

**KO**, knockout

**MS**, mass spectrometry

**MTT**, 3‐(4,5‐dimethylthiazol‐2‐yl)‐2,5‐diphenyltetrazolium bromide

**NanoBiT**, NanoLuc binary technology

**OCR**, oxygen consumption rate

**PBMC**, peripheral blood mononuclear cell

**PCA**, principal component analysis

**PMNC**, polymorphonuclear cell

**PSC**, primary sclerosing cholangitis

**RNA‐seq**, RNA sequencing

**SNP**, single‐nucleotide polymorphism

**TGFP**, TurboGFP

**WT**, wild‐type

G protein‐coupled receptor 35 (GPR35) has emerged as a receptor of significant interest in several physiological processes, due to widespread expression in the gastrointestinal tract, immune cells, cardiovascular system and central nervous system. Moreover, GPR35 biology has been associated with pathologies of the respective tissues, including chronic inflammatory diseases of the gastrointestinal tract [1], ischemic heart disease [2], and cancer of the lung [3], breast [4], stomach [5], and colon [6].

GPR35 is still considered an orphan GPCR [7], since suggested endogenous ligand molecules, such as kynurenic acid, the chemokine CXCL17, and 5‐HIAA are disputed [8, 9, 10]. In contrast, a number of synthetic ligands have been identified, including mast cell stabilizers cromolyn and lodoxamide, as well as PDE5 inhibitor zaprinast and pamoic acid, a counterion for drug formulations [11, 12, 13, 14]. One of the intriguing aspects of the *GPR35* gene is the potential to generate multiple protein isoforms, which arise from alternative splicing events that yield different transcripts (Fig. 1). Transcript 1 is the shortest and consists of Exons 6 and 7. Transcripts 2–4 are very similar in sequence and length but are approximately 1000 nucleotides longer than Transcript 1 and consist of Exons 1, 2, 3, 4, 5, and 7. It has been repeatedly hypothesized that Transcripts 2–4 translate into a long GPR35 isoform that is produced via usage of an alternative start codon in Exon 5, leading to an additional 31 amino acids on the N‐terminal extracellular part of conventional short GPR35 [12, 15, 16].

**Fig. 1:**
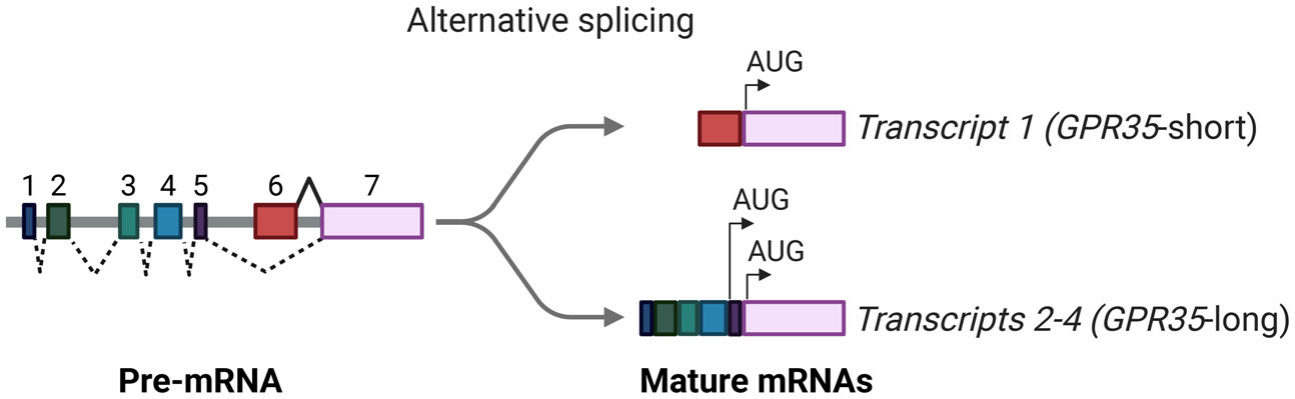
Alternative splicing of the human *GPR35* gene generates 4 transcripts that lead to *GPR35*‐short and *GPR35*‐long isoforms. The *GPR35* gene consists of seven exons. Transcript 1 (3300 nt) consists of Exons 6 and 7. Transcripts 2–4 (approx. 4100 nt) consist of Exons 1, 2, 3, 4, 5, and 7. Exon 5 contains an alternative start codon (AUG).

Increased expression of *GPR35* has been observed in several cancers, including colorectal, gastric, and breast cancers [4, 6, 16]. Genome‐wide association studies (GWAS) have identified several *GPR35* risk variants that are associated with inflammatory bowel disease (IBD) and primary sclerosing cholangitis (PSC) [17, 18, 19, 20, 21], diseases that exhibit a significant propensity for the development of colorectal cancer (CRC) and cholangiocarcinoma (CCA), respectively. The coding variant rs3749171 has been previously shown to lead to increased activity of the GPR35 receptor by driving metabolism and proliferation *in vitro*, suggesting a potential pathogenic role in IBD‐ and PSC‐associated cancer [22]. Understanding which isoforms are upregulated in cancer will provide valuable insights into the molecular underpinnings of cancer progression. Finally, targeting a specific oncogenic GPR35 protein isoform could provide a promising avenue for cancer therapy without affecting the normal physiological functions of GPR35 in other tissues. While the present study does not address tumor initiation *in vivo*, it focuses on defining epithelial‐specific *GPR35* isoform expression and functional phenotypes relevant to tumor growth and metabolism.

## Materials and methods

### Cell lines and culture conditions

HEK‐293 (RRID:CVCL_0045), Caco‐2 (RRID:CVCL_0025), THP‐1 (RRID:CVCL_0006), HT‐29 (RRID:CVCL_0320), and EGI‐1 (RRID:CVCL_1193) were obtained from repository sources (DSMZ) with supplier‐provided short tandem repeat (STR) authentication; no additional in‐house STR profiling was performed. HuCC‐T1 (RRID:CVCL_0324) and Mz‐ChA‐1 (RRID:CVCL_6932) were obtained from an originating laboratory (Dr. Gore's Lab at Mayo Clinic, Rochester, MN, USA) and were not independently STR‐authenticated. All cell lines were routinely tested for mycoplasma contamination and confirmed negative throughout the study. HEK‐293 cells were cultured in Minimum Essential Medium, MEM(1×) + GlutaMAXTM‐I (Gibco, Waltham, MA, USA) with 10% fetal bovine serum (FBS) and 1× penicillin/streptomycin (P/S). Caco‐2 cells were cultured in VWR® Tissue Culture Flasks (AvantorTM, Radnor, PA, USA) in Minimum Essential Medium Eagle (Sigma‐Aldrich, St. Louis, MO, USA) supplied with 20% FBS, 1× P/S and 2 mm L‐glutamine (Gibco). THP‐1 cells were cultured in high glucose DMEM supplied with 10% FBS, 1× P/S and 0.5 mm mercaptoethanol. HT‐29 and EGI‐1 cells were cultured in RPMI1640 supplied with 10% FBS, 1× P/S and 2 mm L‐glutamine (Gibco). HuCCT‐1 and Mz‐ChA‐1 cells were cultured in RPMI1640 supplied with 10% FBS, 1× P/S and 2 mm L‐glutamine (Gibco). All cell lines were incubated at 37 °C in a humidified incubator (5% CO2) (VWR). The morphology and confluence of the cells were assessed under a light microscope (Olympus CKX41).

### Immunoprecipitation

HEK‐293 T cells were transiently transfected with TGFP or Flag‐tagged constructs. After 24 h, cells were lysed in NP‐40 lysis buffer (50 mm Tris, pH 7.4, 250 mm NaCl, 5 mm EDTA, 1% NP‐40) containing Halt Protease Inhibitor Cocktail and Phosphatase Inhibitor (Thermo Fisher Scientific, Waltham, MA, USA). The lysates were centrifuged for 10 min at 10 000 ***g*** at 4 °C. The supernatants were collected and protein concentration was measured using the bicinchoninic acid (BCA) method (Thermo Fisher Scientific). Protein concentration was normalized across samples. Protein was precipitated with TGFP Antibody (AB5134; Evrogen, Moscow, Russia) or Flag Antibody (ab205606; Abcam, Cambridge, UK). Precipitation was carried out overnight at 4 °C under constant shaking. IgG Isotype control antibody was used as a control. Protein‐Antibody complexes were then conjugated to Protein A/G sepharose beads (ThermoFisher) each for 1 h at 4 °C under constant shaking.

### On‐beads digestion

The beads were resuspended in 0.2% ProteaseMax Surfactant (Promega, Madison, WI, USA) in 50 mm NH_4_HCO_3_ and were further diluted with 93.5 μL 50 mm NH_4_HCO_3_. The proteins were reduced in 10 mm DTT at 56°C for 30 min and alkylated in 15 mm iodoacetamide for 20 min at room temperature. One microliter of 1% ProteaseMax Surfactant and 1.8 μL 1 μg·μL^−1^ of trypsin (Promega) or LysC (Wako, Osaka, Japan) were added to each sample for overnight on‐beads protein digestion at 37 °C. The resulting peptides were desalted by reversed‐phase chromatography using C18 microcolumns prepared by stacking three layers of C18 Empore Extraction Disk (Varian, St. Paul, MN) into 200‐μL pipette tips.

### Mass spectrometry

The desalted samples were analyzed by Easy nLC1000 nano‐LC system connected to a quadrupole—Orbitrap (QExactive) mass spectrometer (Thermo Electron, Bremen, Germany) equipped with a nano electrospray ion source (EasySpray/Thermo). For liquid chromatography separation, we used an EasySpray column (C18, 2 μm beads, 100 Å, 75 μm inner diameter) (Thermo) capillary of 25 cm bed length. The flow rate used was 0.3 μL·min^−1^, and the solvent gradient was 2% to 7% in 5 min and then to 30% B in 60 min. The column was finally washed in 90% B wash for 20 min. Solvent A was aqueous 0.1% formic acid, whereas solvent B was 100% acetonitrile in 0.1% formic acid. Column temperature was kept at 60 °C.

The mass spectrometer was operated in the data‐dependent mode to automatically switch between MS and MS/MS acquisition. Survey full scan MS spectra (from m/z 300 to 1750) were acquired in the Orbitrap with resolution *R* = 60 000 at m/z 200 (after accumulation to a target of 3 000 000 ions in the quadruple). The method used allowed sequential isolation of the most intense multiply charged ions, up to 10, depending on signal intensity, for fragmentation in the HCD cell using high‐energy collision dissociation at a target value of 100 000 charges or maximum acquisition time of 20 ms. MS/MS scans were collected at 15 000 resolution at the Orbitrap cell. Target ions already selected for MS/MS were dynamically excluded for 30 s. General mass spectrometry conditions were: electrospray voltage, 2.1 kV; no sheath and auxiliary gas flow, heated capillary temperature of 250 °C, normalized HCD collision energy 25%.

### Protein identification and label‐free quantitation

MS raw files were submitted to the maxquant software version 1.6.1.0 (Cox J and Mann M, Nature Biotechnology 26, 1367–1372, 2008) for protein identification. Parameters were set as follows: carbamidomethylation as fixed modification and protein N‐acetylation and methionine oxidation as variable modifications. First search error window of 20 ppm and main search error of 4.5 ppm. Trypsin without proline restriction or LysC enzyme option was used, with two allowed miscleavages. Minimal unique peptides were set to 1, and false discovery rate (FDR) allowed was 0.01 (1%) for peptide and protein identification. The reviewed Uniprot human database (downloaded October 2017) was used with Q9HC97‐2 and GFP added. Generation of reversed sequences was selected to assign FDR rates.

### Human samples

Cholangiocytes were obtained from the bile ducts of patients undergoing ERCP, recruited from the Section of Gastroenterology at the Department of Transplantation Medicine, Oslo University Hospital Rikshospitalet (Approved project ‘Functional characterization of disease genes in primary sclerosing cholangitis’, REK 2012/286). Written consent was obtained from all study participants before sampling, in accordance with the Declarations of Helsinki and Istanbul. Colon biopsies were collected from 9 subjects with cancer as part of a project on mechanisms of CRC, approved by the regional Committees for Medical and Health Research Ethics of Southeast Norway (REK 2018/703). All patients provided written informed consent.

### Antibody generation

A custom isoform‐specific antibody was generated by Davids Biotechnology (Regensburg, Germany) using rabbit immunization. Recombinant GPR35‐short and GPR35‐long proteins were expressed in *E. coli* as described previously [23]. Antibody specificity for GPR35‐long was assessed by ELISA using the N‐terminal peptide unique to GPR35‐long as well as a C‐terminal peptide shared by both isoforms. Sera showing selective reactivity to the GPR35‐long N‐terminal peptide but not to the shared C‐terminal peptide were selected for downstream applications.

### Immunohistochemistry

Formalin‐fixed and paraffin embedded sections cut at 4 μm were cleared with xylene and dehydrated in ethanol. Antigen retrieval was performed using citrate or EDTA buffer for 10 min at sub‐boiling temperature followed by blocking of endogenous peroxidase activity. Primary antibody was incubated overnight at 4 °C and secondary biotinylated antibody was detected with streptavidin‐HRP (Vector Laboratories, Newark, CA, USA). Sections were developed using a DAB Peroxidase Substrate kit (Vector Laboratories).

### Western blot

Whole‐cell lysates were prepared by lysing cells in NP‐40 lysis buffer supplemented with protease inhibitors, followed by clarification by centrifugation at 10 000× ***g*** for 10 min at 4 °C. Protein concentration was determined using the BCA assay and equal amounts of protein were subjected to SDS/PAGE under reducing conditions. Where indicated, lysates were deglycosylated prior to electrophoresis according to the manufacturer's instructions. Proteins were transferred to PVDF membranes, which were subsequently blocked in 5% nonfat dry milk in Tris‐buffered saline containing 0.1% Tween‐20 (TBST). Membranes were incubated with primary antibodies overnight at 4 °C, washed with TBST, and incubated with HRP‐conjugated secondary antibodies. Protein bands were visualized using enhanced chemiluminescence and imaged using a digital imaging system.

### Immunofluorescence

Cells grown on glass coverslips were fixed in ice‐cold methanol, washed with phosphate‐buffered saline (PBS), and blocked in PBS containing 1–5% bovine serum albumin. Coverslips were incubated with primary antibodies diluted in blocking buffer, followed by washing and incubation with fluorophore‐conjugated secondary antibodies. Nuclei were counterstained with DAPI. Coverslips were mounted using antifade mounting medium and imaged using a fluorescence microscope. Images were processed using the standard image analysis software, and identical acquisition settings were applied across experimental conditions.

### PBMC isolation

The isolation of peripheral blood mononuclear cells (PBMCs) was performed using a density gradient centrifugation method with Ficoll‐Paque. Peripheral blood samples were collected from healthy donors following informed consent and according to the ethical guidelines approved by. Blood was collected into tubes containing anticoagulant to prevent clotting. Ficoll‐Paque PLUS (GE Healthcare, Chicago, IL, USA) was prepared and equilibrated to room temperature before use. Phosphate‐buffered saline without calcium and magnesium was prepared and kept on ice. Collected blood was diluted 1 : 1 with PBS to reduce viscosity and facilitate layering over Ficoll‐Paque. Diluted blood was carefully layered over an equal volume of Ficoll‐Paque in a 50 mL conical tube, ensuring minimal mixing at the interface. Tubes were centrifuged at 400× ***g*** for 30 min at room temperature with the brake off. The PBMC layer was carefully collected using a sterile pipette and transferred to a new 50‐mL conical tube. Collected PBMCs were washed by adding PBS to a total volume of 50 mL and centrifuged at 300× ***g*** for 10 min at room temperature. The supernatant was discarded, and the cell pellet was resuspended in PBS. This washing step was repeated twice. The final cell pellet was resuspended in PBS for downstream applications.

### Cholangiocyte isolation

ERCP brush samples were placed into William's E+ (WE+) medium (Gibco Inc., Waltham, Massachusetts, USA) with 50 ng·mL^−1^ of epidermal growth factor (EGF) (R&D Systems Inc., Minneapolis, Minnesota, USA) and 10 μm Rho‐associated kinase inhibitor Y‐27632 (Selleck Chemicals LLC., Houston, Texas, USA) on ice and carefully washed to collect patient material. Isolated ERCP material was centrifuged at 400 ***g*** for 4 min; supernatant was discarded, and the cells were seeded onto 24‐well cell culture plates in 50 μL droplets of supplemented WE + culture medium [24] and Matrigel (Corning Inc., New York, New York, USA) mixed at a 1 : 3 ratio for *in vitro* 3D expansion [25].

### RNA extraction and RT‐qPCR

RNA was isolated using the SV Total RNA Isolation System (Promega). RNA was then reverse transcribed with RevertAid First Strand cDNA Synthesis Kit (ThermoFisher), and FastStart Universal SYBR Green Master (Rox) (MERCK) qPCR was performed using MX‐3000 (Agilent Technologies, Santa Clara, CA, USA). Target gene expression is expressed as a ratio to housekeeping gene expression. For oligonucleotide sequences, see Table 1.

**Table 1:** Oligonucleotides.

Item ID	Name	Target	Sequence	Application note
LR‐20220919‐131	qORF_Gpr35AB_rev	GPR35	TAGAAGCCCAGCTTGATCGC	Transcripts 1–4
LR‐20220919‐163	qhgpr35b_fwd	GPR35	CCTTGCGTCTCTCTGACCAT	Transcripts 2–4
LR‐20230110‐166	EcoRI‐GPR35ref‐R	GPR35	CGGAATTCTAGGCGAGGGTCACGCACAG	
LR‐20220919‐213	gpr35_gRNA63‐S	GPR35	CACCGtggcccccagcgatcaagct	
LR‐20220919‐185	gpr35_gRNA747‐S	GPR35	CACCGcgcgacggatcgtctccagg	
LR‐20220919‐178	lacZ‐F	lacZ	ggctacgaagacagattaATGTGAGTTAGCTCACTC	
LR‐20220919‐156	KpnI_gpr35a‐F	GPR35	GAGGGTACCCATGAATGGCACCTACAACACCTG	
LR‐20230110‐164	qhgpr35a‐r	GPR35	GGTGCCATTCATGGTCCTGC	GPR35 transcript 1 specific
LR‐20220919‐122	GAPDH‐378404907c1‐R human	GAPDH	GGCTGTTGTCATACTTCTCATGG	
LR‐20220919‐231	SgfI_GPR35A_F	GPR35	CCGGCGATCGCATGAATGGCACCTACAACACCTG	
LR‐20220919‐127	gpr35_747Left	GPR35	CTTCTGCTCCCTGAAGGTGG	
LR‐20220919‐192	GPR35_236Right	GPR35	caccttcagggagcagaagacca	
LR‐20230110‐161	XhoI‐GPR35‐R	GPR35	CGCTCGAGGGCGAGGGTCACGCACA	pET28a‐Mistic cloning
LR‐20220919‐191	Sgfl_GPR35B_F	GPR35	CGGGCGATCGCATGCTGAGTGGTTCCCGG	
LR‐20220919‐150	U6prom‐F		CGTAACTTGAAAGTATTTCGATTTCTTGGC	
LR‐20220919‐214	gpr35_gRNA236‐A	GPR35	AAACtgcgagacacctcagacacgC	
LR‐20220919‐130	Sgfl_gpr35a_fwd	GPR35	GAGgcgatcgcCATGAATGGCACCTACAACACCTG	
LR‐20220919‐162	hgpr35ies‐R	GPR35	GACAGGAGATTGGCAGGCAT	
LR‐20230110‐163	qhgpr35a‐f	GPR35	CCCTTCTCAGACAGCCACTG	GPR35 transcript 1 specific
LR‐20220919‐217	gpr35_gRNA236‐S	GPR35	CACCGcgtgtctgaggtgtctcgca	
LR‐20220919‐132	gpr35_236Right	GPR35	AGGTAGATGCCCTGGGAGAG	
LR‐20220919‐198	qhgpr35b_rev	GPR35	CCAAGTAGGCGTAGAAGCCC	Transcripts 2–4
LR‐20220919‐153	gpr35_63Left	GPR35	CACCTACAACACCTGTGGCT	
LR‐20220919‐221	BbsI_Z‐R		CTAAAACAGGTCTTCAACTATGCGGCATCAGAG	
LR‐20220919‐209	GAPDH‐378404907c1‐F human	GAPDH	GGAGCGAGATCCCTCCAAAAT	
LR‐20220919‐216	hgpr35res‐F	GPR35	CCCTTCTCAGACAGCCACTG	
LR‐20220919‐205	XhoI_GPR35_R	GPR35	CGCTCGAGGGCGAGGGTCACGCACA	
LR‐20230110‐160	EcoRI‐GPR35ref‐F	GPR35	CGGAATTCATGAATGGCACCTACAACACCTG	pET28a‐Mistic cloning
LR‐20220919‐129	BamHI_gpr35‐R	GPR35	GCGGGATCCGGCGAGGGTCACGCACA	
LR‐20220919‐234	gpr35_gRNA747‐S	GPR35	AAACcctggagacgatccgtcgcgC	
LR‐20220919‐157	GPR35_236Left	GPR35	gaatggcacctacaacacctgt	
LR‐20220919‐151	KpnI_A‐F		GAGGGTACCATGAATGGCACCTACAACAC	
LR‐20220919‐136	BbsI_Z‐F		CGAAACACCGGGTCTTCATGTGAGTTAGCTCACTCATTAG	
LR‐20220919‐232	lacZ‐R	lacZ	ggctacgaagacagtaatAACTATGCGGCATCAGAG	
LR‐20220919‐171	gpr35_63Right	GPR35	AGGTAGATGCCCTGGGAGAG	
LR‐20220919‐146	gpr35_236Left	GPR35	CACCTACAACACCTGTGGCT	
LR‐20220919‐222	Mlul_FLAG_gpr35_rev	GPR35	GCGacgcgtTTACTTGTCGTCATCGTCTTTGTAGTCGGCGAGGGTCACGCACA	
LR‐20220919‐226	gpr35_gRNA63‐A	GPR35	AAACagcttgatcgctgggggccaC	
LR‐20220919‐218	hgpr35res‐R	GPR35	GGTGCCATTCATGGTCCTGC	
LR‐20220919‐194	KpnI_gpr35b‐F	GPR35	GAGGGTACCCATGCTGAGTGGTTCCCGG	
LR‐20220919‐208	qORF_Gpr35A_fwd	GPR35	TGAATGGCACCTACAACACCT	Transcripts 1–4
LR‐20230110‐150	SgfI_mGpr35_F		GTAGCGATCGCATGAATAGTACAACCTGTAACAGCACCC	
LR‐20230110‐151	MluI_3XFlagrGpr35_R		GTAACGCGTTTACTTGTCATCGTCATCCTTGTAGTCGATGTCATGATCTTTATAATCACCGTCATGGTCTTTGTAGTCGGTGAGGCTCAGGCTCTG	
LR‐20220919‐123	mut‐R	GPR35	5′ d Phos‐CGAAGGGCAAGGTGCACAGCAG	
LR‐20220919‐175	gpr35_747Right	GPR35	GCACAGAGAGTCCTGGCTTT	
LR‐20220919‐236	Sgfl_gpr35b_fwd	GPR35	GAGgcgatcgcCATGCTGAGTGGTTCCCGG	
LR‐20230110‐162	EcoRI‐GPR35iso‐F	GPR35	CGGAATTCATGCTGAGTGGTTCCCGGGC	pET28a‐Mistic cloning
LR‐20220919‐135	qORF_Gpr35B_fwd	GPR35	GTCCTTGCGTCTCTCTGACC	Transcripts 2–4
LR‐20220919‐160	Mlul_gpr35_rev	GPR35	GCGacgcgtGGCGAGGGTCACGCACA	

### *GPR35* cloning and stable cell line generation

The human *GPR35*‐short CDS was obtained from Origene clone RG215174 (Table 2), human *GPR35*‐long CDS from Caco‐2 cells via PCR (Table 1). Primers with MluI and SgfI (1 and 2 in Table 1, respectively) linker sequences were used to amplify the *GPR35* coding sequence. After double digestion of the resulting PCR fragment with MluI and SgfI, the DNA was cloned into the Origene clone RG215174 (Table 2). This resulted in a C‐terminal GFP‐tagged *GPR35* expression system of *GPR35*‐short and *GPR35*‐long.

**Table 2:** Plasmids.

Item ID	Name	ORFs	Tags
LR‐20221025‐25	236pX458	gRNA, Cas9	EGFP
LR‐20221025‐42	pGPR35AGFP	GPR35 reference	TurboGFP
LR‐20221025‐26	63pX458	gRNA, Cas9	EGFP
LR‐20221025‐18S	ARRB2‐LgBiT	Arrestin B2	C‐terminal Small BiT (Arrestin)
LR‐20221025‐41	pGPR35BGFP	GPR35 isoform 1	TurboGFP
LR‐20221025‐8	pBiT2.3‐C_GPR35B	GPR35 isoform 1	Large BiT (Arrestin)
LR‐20221025‐27	747pX458	gRNA, Cas9	EGFP
LR‐20221025‐16	SmBiT‐ARRB2	Arrestin B2	N‐terminal Small BiT (Arrestin)
LR‐20221025‐19	LgBiT‐ARRB2	Arrestin B2	N‐terminal Large BiT (Arrestin)
LR‐20221025‐12	pBiT2.3‐C_GPR35A	GPR35 reference	Large BiT (Arrestin)
LR‐20221025‐13	pBiT1.3‐C_GPR35A	GPR35 reference	Small BiT (Arrestin)
LR‐20221025‐14	pBiT1.3‐C_GPR35B	GPR35 isoform 1	Small BiT (Arrestin)
LR‐20221025‐5	pGPR35R3XFlag	GPR35 reference	3X Flag

We generated Flag‐tagged constructs for the *GPR35*‐short and *GPR35*‐long. Primers with MluI and SgfI (2 and 2 in Table 1, respectively) linker sequences were used to amplify the *GPR35* coding sequence. After double digestion of the resulting PCR fragment with MluI and SgfI, the DNA was cloned into the Origene clone RG215174 (Table 2). This resulted in a C‐terminal Flag‐tagged *GPR35* expression system of reference and isoform 1.

To generate fusion proteins to use in a beta‐arrestin‐2 recruitment assay, plasmid cloning by PCR was conducted. The plasmids used in this study are shown in Table 2. Human *GPR35*‐short and *GPR35*‐long were cloned together with the pBiT 2.3‐C plasmid (SmBit) from NanoBiT, Nano‐Luc Luciferase (Promega), resulting in GPR35a‐ pBiT2.3‐C (GPR35a‐SmBiT) and GPR35b‐pBiT2.3‐C (GPR35b‐SmBiT). Beta‐arrestin 2 (ARRB2) was cloned together with the pBiT 1.3‐C plasmid (LgBiT) in two different conformations: ARRB2‐ pBiT1.3‐C (ARRB2‐LgBiT) and pBiT1.3‐C‐ARRB2 (LgBiT‐ARRB2). All expression vectors were cloned and stored in DH5alpha. All constructs were verified by Sanger sequencing.

### Transfection of cell lines and selection of stable clones

Cells were plated in Nunc delta 6‐well plate (Thermo Scientific) 1 day before transfection. The next day, the plasmids were transiently transfected into HEK‐293 cells using EscortTM IV Transfection Reagent (Sigma‐Aldrich) following the manufacturer's protocol. Cells were grown overnight in an incubator at 37 °C, 5% CO_2_. As a transfection control, pmaxGFP (Lonza, Basel, Switzerland) was used. The transfection efficiency was evaluated under a microscope (Nikon Eclipse TS100, Nikon, Tokyo, Japan) by observing green fluorescent protein (GFP) in the cells. Selection of stable cell lines expressing TGFP‐ and Flag‐tagged *GPR35* was accomplished by growing HEK‐293 cells in the presence of 400 μg·mL^−1^ G418 (Sigma Merck) for 10 days and subsequent colony picking to obtain clonal cultures.

### Transcriptomic data

Sequencing data for quantification of isoform‐specific abundance of *GPR35* transcripts were obtained from ArrayExpress (https://www.ebi.ac.uk/arrayexpress) with accession numbers E‐MTAB‐9543, E‐MTAB‐9536, E‐MTAB‐9532, E‐MTAB‐9533, and E‐MTAB‐10386 [26]. The 10X sequencing data for quantification of *GPR35* transcripts in subjects with CRC were obtained from GEO: GSE178341 [27].

### Transcriptomic analysis of *GPR35* ‐expressing and *GPR35* ‐deficient HEK‐293 and Caco‐2 cells

To assess the transcriptomic effects of *GPR35* isoform overexpression, stably expressing *GPR35* short‐TGFP and *GPR35* long‐TGFP, and parental (Control) HEK‐293 cells were used. To assess the transcriptomic effects of *GPR35* deletion and ligand stimulation, wild‐type (WT) and *GPR35* knockout (KO) Caco‐2 cells were treated with vehicle or the GPR35 agonist lodoxamide for 6 h. Total RNA was extracted using a standard column‐based purification method, and RNA integrity was confirmed prior to library preparation. RNA‐seq libraries were generated using poly‐A selection and sequenced on an Illumina platform to obtain paired‐end reads. HEK‐293 cells were sequenced on Illumina NovaSeq 6000 (2 × 150 bp paired‐end), Caco‐2 cells on Illumina NovaSeq X Plus (2 × 50 bp paired‐end) at the Norwegian Sequencing Centre (Oslo, Norway). Raw reads were quality trimmed and adaptor trimmed using bbduk version 39.01 (BBTools; https://jgi.doe.gov/data‐and‐tools/bbtools/) (parameters: ktrim = r k = 23 mink = 11 hdist = 1 tbo tpe qtrim = r trimq = 15 minlen = 36 maq = 15 trimpolya = 10). A decoy‐aware index for the transcript quantificationtool Salmon version 1.10 was built (parameters: ‐‐kmerLen 25) based the human transcriptome (ftp://ftp.ebi.ac.uk/pub/databases/gencode/Gencode_human/release_45/gencode.v45.transcripts.fa.gz) with the full human genome (ftp://ftp.ebi.ac.uk/pub/databases/gencode/Gencode_human/release_45/GRCh38.primary_assembly.genome.fa.gz) as decoy (as described on https://salmon.readthedocs.io/en/latest/salmon.html). Transcript quantification was then performed with Salmon. Transcript abundances were summarized to gene level with R package tximeta version 1.26.2 and imported as a DESeqDataSet object with R package DEseq2 version 1.48.2. Filtered to genes in which > 2 samples (6 for CACO) have > 10 counts. Ensembl gene names and annotation was acquired with R package biomaRt 2.62 [28, 29, 30, 31]. Differential gene expression was assessed using DESeq2. Principal component analysis (PCA) was used to visualize global transcriptomic variation. Shrunken log_2_ fold change was estimated using the ashr package before visualization of significant transcriptional changes by volcano plots [32]. Gene set enrichment analysis (GSEA) and enrichment analysis were performed with clusterprofiler. Prior to functional transcriptomics analysis of *GPR35* overexpression in HEK‐293, *GPR35* was removed from the gene list. This filtering was not performed when comparing WT and KO Caco‐2 cells. GSEA was performed on preranked gene lists against Gene Ontology Biological Processes and Reactome gene sets. Gene sets with fewer than 5 or more than 1000 genes were excluded from the analysis. Genes were ranked by taking their ‐log_10_ *P*‐value and assigning the sign of the log_2_ fold change. The Gene Ontology and Reactome gene sets were downloaded from the Human Molecular Signature Database (MSigDB). Alternative GSEA ranking was based on log_2_ fold change alone. Enrichment analysis was performed on filtered gene lists. All genes with a log_2_ fold change greater than 1 were assigned to the upregulated gene list. Similar, all genes with a log_2_ fold change smaller than −1 were assigned to the downregulated gene list. Pathways with fewer than 5 genes or more than 1000 genes were excluded from the analysis. All transcriptomic analyses were conducted using R and Bioconductor packages.

### CRISPR/Cas9

*GPR35* KO (*GPR35* ^−/−^) Caco‐2 cells were generated by CRISPR/Cas9. Different combinations of gRNA that target *GPR35* Exon 7 were used to induce three independent deletions within Exon 7. To control for potential off‐target effects, the composite GFP, gRNA, and Cas9 expressing plasmid was introduced by transient transfection using Escort IV (Sigma). Mock gRNA was used to obtain *GPR35* WT cells for downstream analysis that have gone through equal treatment. Two days after transfection, successfully transfected cells were identified by GFP expression and subjected to single‐cell sorting followed by clonal expansion. The genotype of the established clones was assessed by PCR and gel electrophoresis. Three biologically independent clonal *GPR35* WT and *GPR35* KO Caco‐2 cell lines were obtained and further expanded for cryopreservation.

### MTT assay

Cell growth was measured by a modified MTT cell culture assay. HEK‐293 cells were seeded in 96‐well tissue culture plates and left to attach overnight. Indicated concentrations of compounds were added and cell growth was analyzed quantitatively via MTT staining. The medium and detached cells were removed by aspiration. 200 μL of PBS buffer were added plus 25 μL of MTT reagent 3‐(4,5‐dimethyl‐thiazol‐2‐yl)‐2,5‐diphenyltetrazolium bromide (5 mg·mL^−1^ in PBS buffer), followed by incubation for 2 h. Afterward, the liquid phase was replaced with 200 μL of a 1 : 25 mixture of 96% acetic acid and 2‐propanol. The reaction product was measured through absorption at 595 nm in triplicate. A595 values were normalized to cells treated with PBS alone.

### Seahorse extracellular flux analysis

Cells were seeded into 96 well XFe Seahorse plates (Agilent) in normal culture medium. 1 h prior to the start of extracellular flux measurements, the medium was changed to Agilent Seahorse XF Base Medium (103334‐100) supplemented with 1 mm L‐glutamine (ThermoFisher) and 10 mm D‐glucose (MERCK). The pH of the medium was set to 7.4 after warming to 37 °C. OCR and ECAR were measured using the XFe96 Seahorse extracellular flux analyzer (Agilent) over the course of 2 h. Oligomycin (1 μm), FCCP (2 μm), and Rotenone/Antimycin A (both 1 μm) (all MERCK) were injected sequentially to allow assessment of respiratory chain functionality.

Basal Respiration: (Last rate measurement before first injection) – (Non‐Mitochondrial Respiration Rate); Maximal Respiration: (Maximum rate measurement after FCCP injection) – (Non‐Mitochondrial Respiration); Nonmitochondrial Oxygen Consumption: Minimum rate measurement after Rotenone/antimycin A injection. Glycolytic function was measured in a separate protocol. Cells were incubated in seahorse assay medium without glucose. Glycolysis was assessed through measurement of ECAR after sequential injections with D‐glucose (10 mm), Oligomycin (1 μm), and 2‐deoxyglucose (100 mm) (Sigma‐Aldrich, UK). Glycolysis: (Last rate measurement before oligomycin injection) – (non‐glycolytic acidification Rate); Maximal glycolytic capacity: (Last rate measurement before glucose injection) – (Last rate measurement before oligomycin injection); Glycolytic reserve: (Last rate measurement before 2‐DG injection) – (Last rate measurement before oligomycin injection).

The Seahorse ATP Rate Assay was employed to measure the rate of ATP production in cellular samples, utilizing the Seahorse XF Analyzer (Agilent Technologies). On the day of the assay, the growth medium was replaced with Seahorse XF Base Medium supplemented with 10 mm glucose, 1 mm pyruvate, and 2 mm glutamine and adjusted to pH 7.4. Cells were washed twice with the assay medium and incubated in a non‐CO_2_ incubator at 37 °C for 1 h prior to the assay. Oligomycin was injected into each well to inhibit mitochondrial ATP synthesis, and changes in OCR and ECAR were recorded. The OCR and ECAR data were analyzed using the seahorse wave software. Mitochondrial ATP production was calculated from the decrease in OCR following oligomycin injection, and glycolytic ATP production was calculated from the increase in ECAR. ATP production rates were normalized to cell number measured at the end of the experiment using Hoechst 33342 staining (10 μm final concentration) of DNA and quantification in a benchtop plate reader at Ex350 nm and EM 461 nm.

### Arrestin assay

The NanoLuc® Binary Technology (NanoBiT) Protein : Protein Interaction System from Promega was used to perform a beta‐arrestin‐2 recruitment assay to explore the activation of *GPR35*‐short and *GPR35*‐long by different compounds. In this study, the respective WT and mutated *GPR35* isoforms were fused together with the Small BiT and beta‐arrestin‐2 with the Large BiT. To be able to examine the protein interactions in living cells, Nano‐Glo® Live Cell Assay System was used. The assay was performed following the manufacturer's protocol, NanoBiT® Protein : Protein Interaction System, with minor modifications. All compounds were analyzed in a minimum of three replicates. The compounds were dissolved in dimethyl sulfoxide (DMSO) to make stock solutions of highest soluble concentration. Zaprinast 100 mm, kynurenic acid 37 mm, pamoic acid 100 mm, lodoxamide 6.4 mm, CID‐2745687 100 mm, and ML145 100 mm. For analysis, the cell plate was analyzed in the synergy H1 (BioTek®) plate reader. Baseline luminescence was measured for 20 min. After 20 min, 10 μL of compounds was added to each well and the cell plate was placed back in the plate reader. Luminescence was measured every 5 min for 1 h to determine maximum peak height and AUC. Maximum peak height over baseline was used to plot dose–response curves in this study.

### Statistical analysis

All statistical analysis was performed using the prism 7 (graphpad) software. The statistical tests used in each experiment are described in the respective figure legends.

## Results

### *GPR35* ‐long is predominantly detected in epithelial tissue and elevated in gastrointestinal tumors

We first sought to determine whether the cancer‐associated upregulation of *GPR35* previously reported in colorectal and other gastrointestinal cancers [4, 6, 16] could be attributed to a specific isoform. To this end, the expression pattern of GPR35 transcripts was characterized across cell lines, publicly available transcriptomic datasets, and a subset of relevant primary cells and tissues.

We used transcript‐specific primers to evaluate transcripts that would either code for *GPR35* short and *GPR35* long and measured quantitatively expression level in human cells and tissue (Fig. 2A). The highest total *GPR35* expression was observed in colon mucosa biopsies and epithelial cell lines, particularly colorectal and CCA cell lines. The *GPR35*‐short related transcript 1 and *GPR35*‐long related transcripts 2–4 were determined to be excluding each other. Transcript 1 was primarily detected in immune cells whereas transcripts 2–4 were predominantly observed in epithelial cells of the gastrointestinal and biliary tract. The translation of the epithelium‐specific transcripts 2–4 into the GPR35‐long protein was assessed by development of an immune precipitation mass spectrometry protocol to measure GPR35‐long protein in cells that express high levels of *GPR35* transcripts 2–4. We overexpressed the *GPR35*‐long CDS with a C‐terminal fusion of *TurboGFP* in HEK‐293 cells to purify GPR35‐long protein via immune precipitation using an anti‐TGFP antibody. The resulting protein was used to develop a shotgun mass spectrometry protocol for detection of the isoform‐specific N‐terminal peptide in native protein samples (Table 3). Using this protocol, we were able to detect a N‐terminal GPR35‐long‐specific peptide in Caco‐2 cells, suggesting that transcripts 2–4 may be translated into an alternative protein isoform (Fig. 2A,B). We then quantified *GPR35* expression in epithelial cells of the colon cancer single‐cell atlas [27] and measured significantly higher levels of total *GPR35* transcripts in biopsies from colorectal tumor tissue compared with normal tissue (Fig. 2C). We developed a GPR35‐long‐specific antibody that would allow the assessment of GPR35‐long protein abundance specifically *in situ*. To validate the specificity of the custom GPR35‐long antibody, we performed immunoblotting and immunofluorescence analyses using heterologous expression of TGFP‐tagged *GPR35*‐short or *GPR35*‐long constructs in HEK‐293 cells. Following deglycosylation, the antibody selectively detected GPR35‐long but not GPR35‐short by western blot, while a pan‐GPR35 antibody recognized both isoforms (Fig. S1A). Consistently, the GPR35‐long antibody did not stain *GPR35*‐short‐expressing cells by immunofluorescence but specifically labeled *GPR35*‐long‐expressing cells (Fig. S1B,C), supporting isoform selectivity in overexpression systems. Consequently, we used these antibodies in immunohistochemistry experiments to investigate colon tissue obtained from colorectal subjects with CRC during resection, including both biopsies containing tumor tissue and neighboring healthy tissue. *GPR35*‐long was expressed substantially higher in epithelium from tumor tissue compared with healthy sections (Fig. 2D). Given the modest number of clinical samples analyzed, these observations should be considered supportive rather than definitive. Although immunohistochemistry alone cannot establish molecular specificity for low‐abundance GPCRs in human tissue, the epithelial‐restricted tumor‐associated staining pattern is supported by multiple orthogonal approaches, including isoform‐specific transcript detection, isoform‐specific peptide identification by mass spectrometry, genetic deletion models, and pharmacological inhibition.

**Fig. 2:**
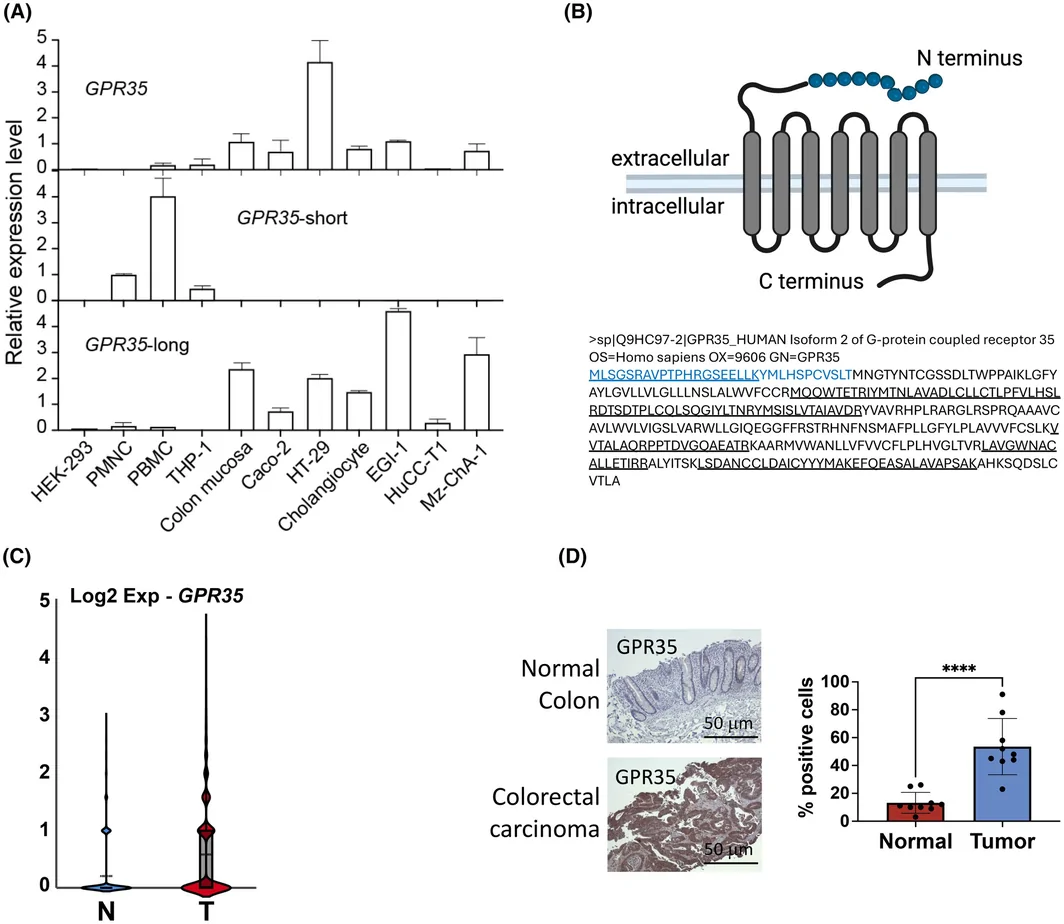
GPR35‐*long* is expressed at elevated levels in gastrointestinal cancer cell lines. (A) Relative expression of all *GPR35* transcripts and *GPR35*‐short‐ and *GPR35‐*long‐specific transcripts in primary human cells and cancer cell lines; human embryonic kidney cell line HEK‐293, PMNC (polymorphonuclear cells), PBMC (peripheral blood mononuclear cells), monocyte cell line THP‐1, colorectal cancer cell lines Caco‐2 and H‐T29, cholangiocarcinoma cell lines EGI‐1, HuCC‐T1 and Mz‐ChA‐1, primary human cholangiocytes and human colon mucosa biopsy tissue. Relative expression was measure by transcript‐specific quantitative RT‐PCR of all annotated *GPR35* transcripts. *N* = 3 technical replicates; no statistical comparison performed. Data represented as mean ± s.e.m. (B) A peptide specific for the GPR35‐long‐specific N‐terminal sequence (in blue) was detected using shotgun mass spectrometry in whole‐cell lysates (WCL) of Caco‐2 cells. GPR35‐derived peptides detected are underlined. (C) *GPR35* transcript abundance in epithelial cells from colorectal tumor (T) and adjacent normal tissue (N) from the colorectal cancer single‐cell atlas [27] (62 patients), analyzed in Loupe Browser (10× Genomics). Differential expression assessed using an exact negative binomial test (sSeq method). (D) Immunohistochemistry staining of GPR35‐long in colon biopsies from subjects with colorectal cancer. GPR35‐long was detected using a proprietary isoform‐specific antibody in mucosal tumor biopsy samples. Samples were obtained during tumor resection and included patient‐matched normal tissue. Slides were scanned and GPR35‐positive staining determined and quantified using the qupath software. Scale bar: 50 μm. *N* = 9. Data represented as mean ± s.e.m. two‐tailed Student's *t*‐test. ****P < 0.0001.

**Table 3:** Shotgun MS Caco‐2.

Sequence	Unique (proteins)	Intensity Trp	Intensity LysC	LFQ intensity Trp	LFQ intensity LysC
MLSGSRAVPTPHRGSEELLK	Yes	0	21189000	0	18174000
DTSDTPLCQLSQGIYLTNR	No	54816000	0	37457000	0
EFQEASALAVAPSAK	No	0	10388000	0	8910000
IYMTNLAVADLCLLCTLPFVLHSLR	No	13407000	0	9161600	0
LAVGWNACALLETIR	No	68473000	0	46789000	0
LAVGWNACALLETIRR	No	3418200	0	2335700	0
LSDANCCLDAICYYYMAK	No	34800000	1239500	23780000	1063100
MQQWTETR	No	32560000	0	22249000	0
VVTALAQRPPTDVGQAEATR	No	47899000	0	32730000	0
YMSISLVTAIAVDR	No	18539000	0	12668000	0

### *GPR35* ‐long overexpression is associated with transcriptional changes linked to cell growth/adhesion and metabolism

Having established that *GPR35*‐long is predominantly expressed in gastrointestinal epithelial cells and its expression increases in colorectal tumors, we next sought to identify the transcriptional programs associated with each isoform. To enable controlled, isoform‐resolved comparison in the absence of endogenous *GPR35* expression, we stably expressed *GPR35*‐short and *GPR35*‐long individually in HEK‐293 cells — a *GPR35*‐negative heterologous system — and performed bulk RNA sequencing. These experiments were designed to identify pathway‐level signatures that distinguish the two isoforms; the resulting transcriptional profiles should be interpreted as reflecting intrinsic isoform‐specific differences in an engineered context rather than direct evidence of endogenous tumor‐cell mechanisms.

C‐terminal TurboGFP (TGFP) and Flag‐tagged *GPR35* CDS, either coding for the short or long isoform, were stably integrated in HEK‐293 cells under the control of the CMV promoter. We confirmed strong and robust overexpression of both isoforms compared with the parental HEK‐293 cell line. We then isolated total mRNA from the respective cell lines to perform bulk RNA sequencing. Additionally, we determined expression levels of *GPR35* across the three conditions: unmodified HEK‐293 cells, HEK‐293 expressing *GPR35*‐short‐*TGFP*, and HEK‐293 expressing *GPR35*‐long‐*TGFP*. Transfecting the long version of *GPR35* resulted in the highest protein expression, indicating isoform‐specific upregulation of *GPR35* (Fig. 3A). The PCA plot of RNA‐seq data demonstrates clear clustering and separation between the three cell populations, suggesting distinct transcriptional profiles (Fig. 3B).

**Fig. 3:**
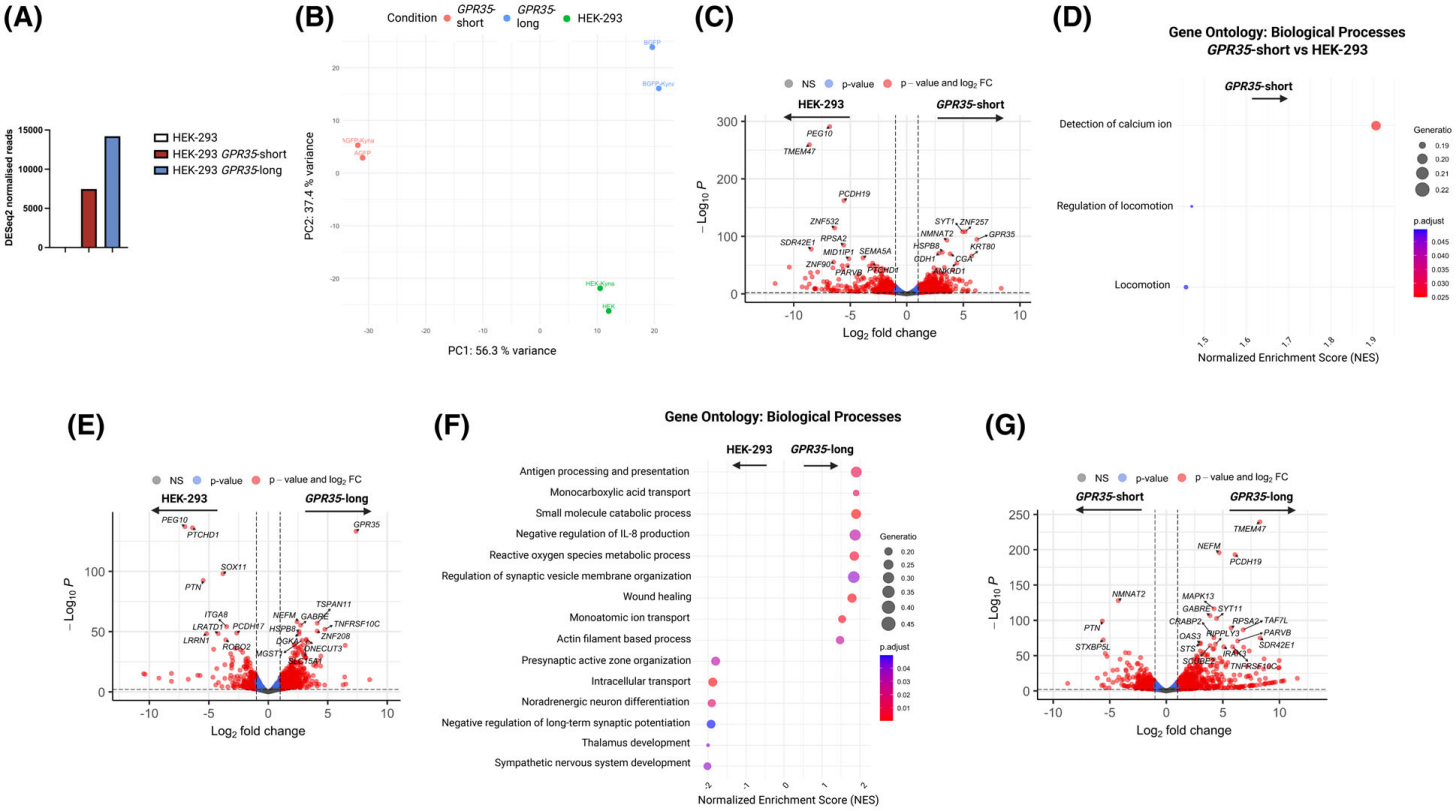
*GPR35*‐long overexpression in HEK‐293 cells is associated with a distinct transcriptional signature compared with *GPR35‐*short. *GPR35*‐negative HEK‐293 cells were transfected with C‐terminal TurboGFP (TGFP)‐ and Flag‐tagged *GPR35*‐short and *GPR35*‐long constructs. Fusion constructs were stably integrated, and cell lines clonally expanded. *N* = 2 individual clones per condition for all experiments. RNA sequencing was performed in duplicate on individual clones and mRNA data subjected to differential gene expression analysis. (A) *GPR35* expression level (normalized reads) of *GPR35*‐negative parental cell line clones, *GPR35*‐short‐ and *GPR35*‐long‐expressing clones. (B) PCA plot of cell lines. *N* = 4 samples per group. (C) Volcano plot showing differentially expressed genes (DEGs) between HEK‐293 expressing *GPR35*‐short and parental cells. Genes with adjusted *P*‐value < 0.05 and absolute log_2_ fold change > 1 are highlighted. (D) Gene set enrichment analysis (GSEA) of significantly altered pathways in *GPR35*‐short‐expressing cells versus HEK‐293 cells. Enriched pathways are plotted based on normalized enrichment score (NES), with point size representing gene ratio and color indicating adjusted *P*‐value. (E) Volcano plot showing differentially expressed genes (DEGs) between HEK‐293 expressing *GPR35*‐long and parental cells. Genes with adjusted *P*‐value < 0.05 and absolute log_2_ fold change > 1 are highlighted. (F) GSEA of significantly altered pathways in *GPR35*‐long‐expressing cells versus HEK‐293 cells. Enriched pathways are plotted based on normalized enrichment score (NES), with point size representing gene ratio and color indicating adjusted *P*‐value. (G) Volcano plot showing differentially expressed genes (DEGs) between HEK‐293 expressing *GPR35*‐long and *GPR35*‐short. Genes with adjusted *P*‐value < 0.05 and absolute log_2_ fold change > 1 are highlighted.

Comparison of *GPR35*‐short‐expressing cells with control HEK‐293 cells reveals a substantial number of differentially expressed genes (DEGs), as shown in the volcano plot with *GPR35* being the most upregulated gene, naturally (Fig. 3C). Gene ontology (GO) enrichment analysis of these DEGs indicates involvement in ‘detection of calcium ion’ and ‘locomotion’ (Fig. 3D). Differentially expressed genes of *GPR35*‐long‐expressing compared with control HEK‐293 cells confirmed *GPR35* being again the most upregulated gene (Fig. 3E). Comparison of *GPR35*‐long‐expressing cells with HEK‐293 reveals a distinct DEG profile in contrast to *GPR35*‐short overexpression, with enrichment of pathways related to, among others, immune signaling, such as ‘antigen processing and presentation’, negative regulation of interleukin 8 production’ and metabolic pathways, such as ‘monocarboxylic acid transport’, ‘small molecule catabolic process’, ‘wound healing’ (Fig. 3F). Direct comparison of *GPR35*‐long and *GPR35*‐short conditions shows robust differential expression (Fig. 3G). However, ranking of DEGs based on *P*‐value detected few altered pathways in enrichment analysis. We re‐analyzed differentially regulated pathways based on log2 fold change and observed upregulation of genes involved in ‘epithelial‐mesenchymal transition’ and ‘ion transport’ in *GPR35*‐long‐expressing cells (Fig. S2A,B). Conversely, enrichment analysis revealed downregulation of genes involved in, among others, locomotion and neuron function in *GPR35*‐long compared with *GPR35*‐short‐expressing cells (Fig. S2C).

Notably, *GPR35*‐long‐specific enrichment of pathways related to monocarboxylic acid transport, small molecule catabolism, and oxidative metabolism predicted a bioenergetic phenotype that we sought to directly test in subsequent functional assays.

### *GPR35* modulates transcriptional programs in intestinal epithelial cells

To determine whether the transcriptional programs associated with *GPR35*‐long overexpression in HEK‐293 cells were relevant in a disease‐appropriate epithelial context, and to assess the consequences of *GPR35* loss rather than gain of function, we developed a *GPR35*‐deficient CRC cell model. *GPR35* was deleted in Caco‐2 cells via CRISPR/Cas9 technology using 3 different gRNAs to target the *GPR35* Exon 7 (930 base pairs in total), leading to partial deletions of CDS base pair positions 63–236, 236–747, and 63–74 and the generation of 3 independent *GPR35* KO (*GPR35* ^−/−^) Caco‐2 cell lines.

To investigate the transcriptional impact of *GPR35* deletion and its interaction with lodoxamide treatment in intestinal epithelial cells, we performed RNA sequencing on *GPR35* KO and WT Caco‐2 cells with or without lodoxamide exposure. Deletion of *GPR35* in Caco‐2 cells resulted in substantial transcriptional changes. Principal component analysis revealed clear separation between WT and *GPR35* KO samples along principal component 1 (PC1), which accounted for 27% of the variance, indicating distinct global gene expression profiles between genotypes (Fig. 4A). These analyses were therefore used to define convergent biological processes for functional validation rather than to infer direct regulatory mechanisms. Differential expression analysis identified numerous significantly altered genes in the WT condition compared with KO (Fig. 4B). The most upregulated transcripts in WT compared with KO cells included *TGFB2*, *SCN9A*, *BDNF*, *C3orf70*, *COL4A4*, *COL4A3*, *FAM43B*, and *GADL1*. Gene set enrichment analysis (GSEA) revealed significant enrichment for pathways related to extracellular matrix (ECM) organization and RNA metabolism in WT compared with KO cells (Fig. 4C). Lodoxamide treatment did not alter the global transcriptional profile of either *GPR35* WT or *GPR35* KO cells (Fig. 3). The upregulation of RNA metabolism pathways in *GPR35* WT cells may partly reflect increased proliferative activity, consistent with the proliferative phenotype observed upon *GPR35*‐long overexpression. Similarly, the enrichment of ECM‐related pathways, including PDGF signaling and integrin interactions in WT cells is consistent with the growth‐associated transcriptional signatures identified in *GPR35*‐long‐overexpressing HEK‐293 cells and connects the loss‐of‐function transcriptional data to the metabolic and proliferative phenotypes described in subsequent sections.

**Fig. 4:**
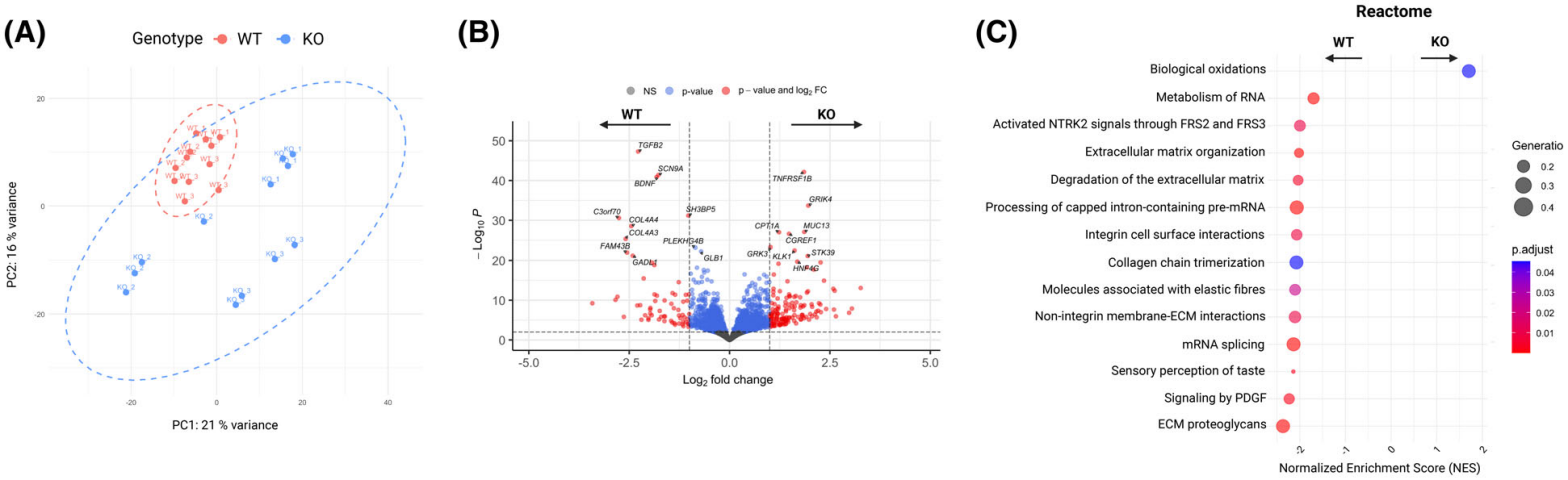
Transcriptomic analysis of *GPR35* knockout (KO) and wild‐type (WT) Caco‐2 cells. (A) Principal component analysis (PCA) of RNA‐seq data from Caco‐2 *GPR35* KO and WT cells without treatment shows distinct clustering based on genotype. Each point represents a biological replicate, colored by genotype (red: WT, blue: KO). Encirclement corresponds to a 95% confidence interval. *N* = 3 biological replicates per genotype. (B) Volcano plot displaying differentially expressed genes (DEGs) in *GPR35* KO versus WT Caco‐2 cells. Genes with significant changes in expression (*P*‐value <0.01 and absolute log_2_ fold change > 1) are highlighted in red, and the top 20 DEGs by adjusted *P*‐value are labeled. (C) Gene set enrichment analysis (GSEA) of significantly altered pathways in *GPR35* KO versus WT Caco‐2 cells. Enriched pathways are plotted based on normalized enrichment score (NES), with point size representing gene ratio and color indicating adjusted *P*‐value.

### *GPR35* ‐long overexpression is associated with increased cellular metabolic activity and growth‐associated phenotypes

The transcriptomic profile of *GPR35*‐long‐expressing HEK‐293 cells was notably enriched for pathways related to oxidative metabolism and small molecule catabolism, suggesting a functional increase in cellular bioenergetics. To directly test this prediction, we performed Seahorse extracellular flux analysis to measure mitochondrial and glycolytic ATP production in *GPR35*‐short‐ and *GPR35*‐long‐expressing HEK‐293 cells.

We expressed the two *GPR35* isoforms individually in *GPR35*‐negative HEK‐293 cells (see Fig. 2A and Fig. 3A). We measured ATP production and observed a significantly increased mitochondrial and glycolytic ATP production rate compared with the *GPR35* negative parental cell line (Fig. 5A). Both mitochondrial and glycolytic ATP production were increased. *GPR35*‐long appeared to specifically increase ATP production compared with *GPR35*‐short. We then verified that both mitochondrial respiration and glycolysis were upregulated upon *GPR35* overexpression. We found that *GPR35*‐long expression resulted in significantly increased oxidative phosphorylation as shown by increased maximal respiration, spare respiratory capacity and mitochondrial ATP production compared with *GPR35*‐short expression (Fig. 5B). Similarly, glycolysis was significantly increased in *GPR35*‐long‐expressing cells compared with *GPR35*‐short (Fig. 5C). Specifically, glycolytic rate, glycolytic reserve and maximal capacity were significantly increased upon *GPR35*‐long expression. Furthermore, we determined that the increase in cellular metabolic activity was associated with the *GPR35* overexpressing cell lines by measuring proliferation using a standard MTT proliferation assay over the course of 5 days. We found that *GPR35*‐long‐expressing cells displayed increased metabolic activity associated with increased proliferation in this assay compared with *GPR35*‐short‐expressing cells (Fig. 6A). This phenotype was observed in all *GPR35*‐long‐expressing cell lines, irrespective of whether TGFP or Flag tag was used for fusion protein construction. We measured fusion protein expression in the tested cell lines using qRT‐PCR and determined variation in TGFP‐tagged *GPR35* expression that was not observed for Flag‐tagged *GPR35*‐short and *GPR3*‐long constructs and did not appear to be associated with the proliferation phenotype (Fig. 6B).

**Fig. 5:**
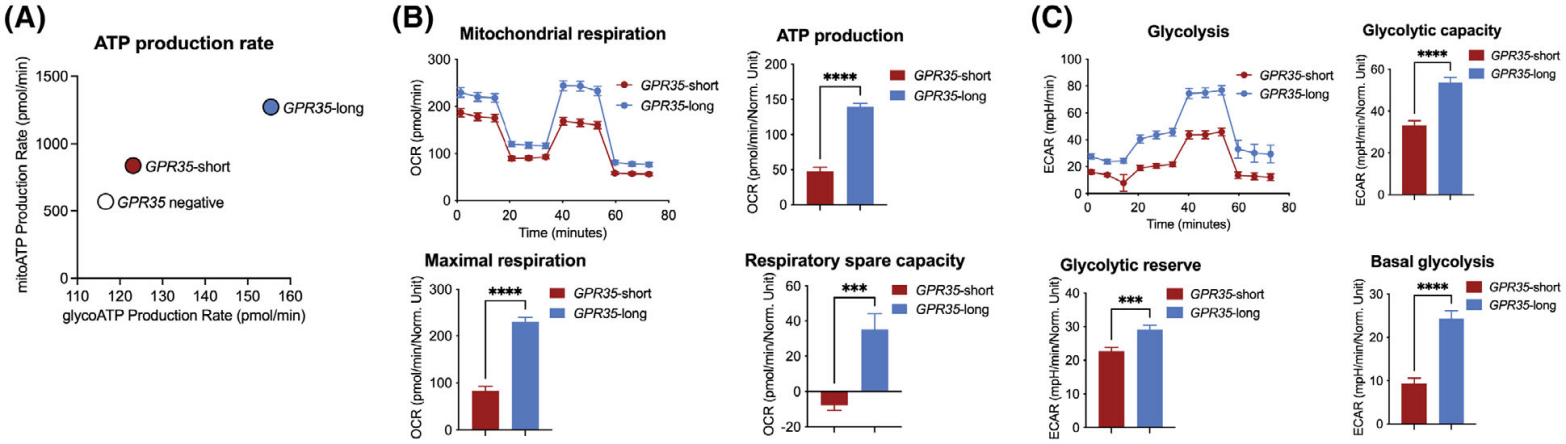
*GPR35*‐long overexpression in HEK‐293 cells is associated with increased aerobic and anaerobic cellular metabolism. The metabolic impact of *GPR35*‐short or *GPR35*‐long expression was analyzed using real‐time metabolic profiling by Seahorse technology. (A) ATP production in *GPR35* expressing cell lines was measured using the Seahorse ATP rate assay. Mitochondrial (mitoATP) and glycolytic (glycoATP) was determined in *GPR35*‐short‐ and *GPR35*‐long‐expressing cell lines compared with *GPR35*‐negative parental cells. (B) Cellular respiration measured as oxygen consumption rate (OCR) using the Seahorse Mito stress test during sequential treatment with oligomycin, FCCP and antimycin/rotenone in *GPR35* expressing HEK‐293 cell clones. Bar charts indicate maximal respiration, ATP production and (respiratory) spare capacity. (C) Glycolytic metabolism of *GPR35* expressing cell lines was analyzed using extracellular acidification rate (ECAR) via the Seahorse Glyco Stress test during sequential injection of oligomycin and 2‐DG. Bar charts show glycolytic rate, glycolytic capacity and glycolytic reserve. *N* = 6 independent experiments for all panels. All data represented as mean ± s.e.m. Statistical significance was calculated by two‐tailed Student's *t*‐test using individual data points for Seahorse assays, *P < 0.05, **P < 0.01, ***P < 0.001, ****P < 0.0001.

**Fig. 6:**
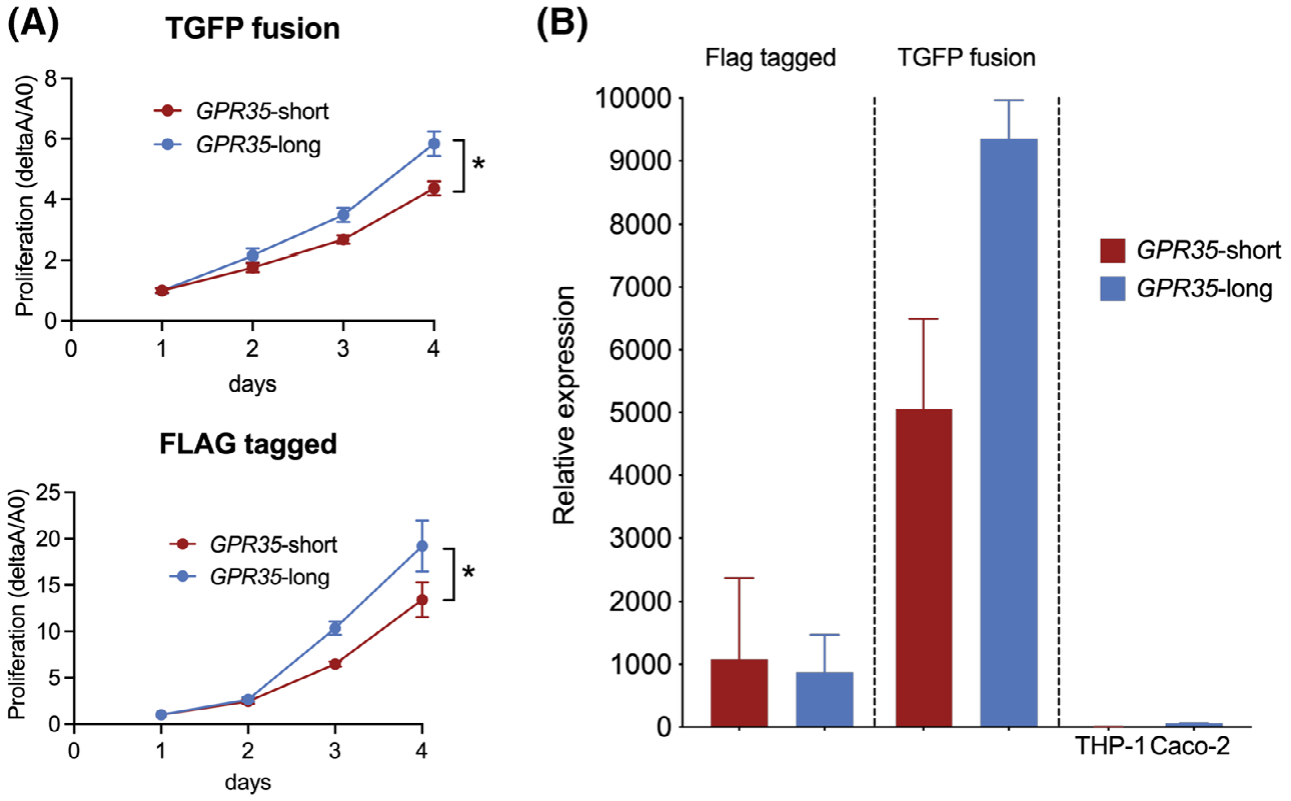
*GPR35*‐long overexpression in HEK‐293 cells is associated with increased proliferation. (A) The impact of GPR35 overexpression on cellular proliferation was analyzed via standard 3‐(4,5‐dimethylthiazol‐2‐yl)‐2,5‐diphenyltetrazolium bromide (MTT) proliferation assay in GPR35‐short or GPR35‐long‐expressing HEK‐293 cells over a period of 5 days. *N* = 6 independent experiments. Area under the curve (AUC) was calculated for each replicate and compared between groups by two‐tailed Student's *t*‐test. Data represented as mean ± s.e.m. *P < 0.05. (B) *GPR35* isoform‐specific expression levels in TGFP‐ and Flag‐tagged clones. Relative expression was measure by quantitative RT‐PCR using specific primers for either *GPR35*‐short and *GPR35*‐long open reading frame. GPR35‐short relative expression levels are referenced to *GPR35* expression level in THP‐1 cells, *GPR35*‐long relative expression levels are referenced to *GPR35* expression level in Caco‐2 cells. *N* = 3. All data represented as mean ± s.e.m. Data are presented as descriptive—no test performed.

### *GPR35* ‐long shows increased constitutive, and ligand‐induced activity compared with reference isoform

The isoform‐specific transcriptional and metabolic phenotypes described above raised the question of what receptor‐level properties distinguish *GPR35*‐long from *GPR35*‐short and could underlie these differences. We therefore characterized the signaling activity of each isoform using a NanoBiT beta‐arrestin 2 recruitment assay in HEK‐293 cells, which provide a *GPR35*‐negative background for controlled isoform comparison independent of epithelial differentiation state.

We used a protein complementation based β‐arrestin assay based on Promega NanoBit technology and fused *GPR35*‐short and *GPR35*‐long individually to SmBit on the C terminus and provided N‐terminally fused LgBit‐arrestin 2 fusion protein as a functional complementation partner to generate luciferase activity measured in live HEK‐293 cells. HEK‐293 cells were used here as a reductionist signaling platform lacking endogenous *GPR35* expression, allowing controlled comparison of isoform‐specific receptor signaling independent of epithelial differentiation state. We compared ligand‐induced GPR35 β‐arrestin 2 coupling in dose–response experiments using GPR35 ligands, kynurenic acid, pamoic acid, zaprinast, and lodoxamide (Fig. 7A,B). We observed dose‐dependent β‐arrestin recruitment by both GPR35 isoforms and recapitulated previously described variations in ligand potency (EC50). There was no significant difference in ligand potency between the GPR35 isoforms for any of the agonists used. Kynurenic acid had the highest EC50 with > 50 μm, whereas lodoxamide activates GPR35 at an EC50 < 20 nm. The other high potency ligands pamoic acid showed nanomolar EC50 values, while zaprinast showed micromolar EC50 values. We observed significantly increased absolute basal as well as maximal luminescence signal levels in cells transfected with *GPR35*‐long compared with *GPR35*‐short, suggesting increased β‐arrestin recruitment (Fig. 7A). In addition, relative β‐arrestin coupling upon ligand binding was increased when cells expressed GPR35‐long compared with GPR35‐short (Fig. 7B). This phenotype was observed for all ligands used except the very low affinity agonist kynurenic acid. We then tested the high potency inhibitors ML145 and CID‐2745687 in the β‐arrestin assay in combination with the high potency agonist lodoxamide. We observed that both ML145 and CID‐2745687 substantially inhibited lodoxamide‐induced β‐arrestin coupling (Fig. 8A). Both antagonists showed slightly more potency toward GPR35‐long (IC_50_ < 400 nm) compared with GPR35‐short (IC_50_ > 1 μM). We then tested ML145 and CID‐2745687 in the absence of agonists. We observed no significant reduction in β‐arrestin coupling for neither GPR35 isoform when using ML145 (Fig. 8B) but determined inverse agonism of CID‐2745687 regarding constitutive GPR35 activity that was significantly pronounced for GPR35‐long (Fig. 8B) compared with GPR35‐short.

**Fig. 7:**
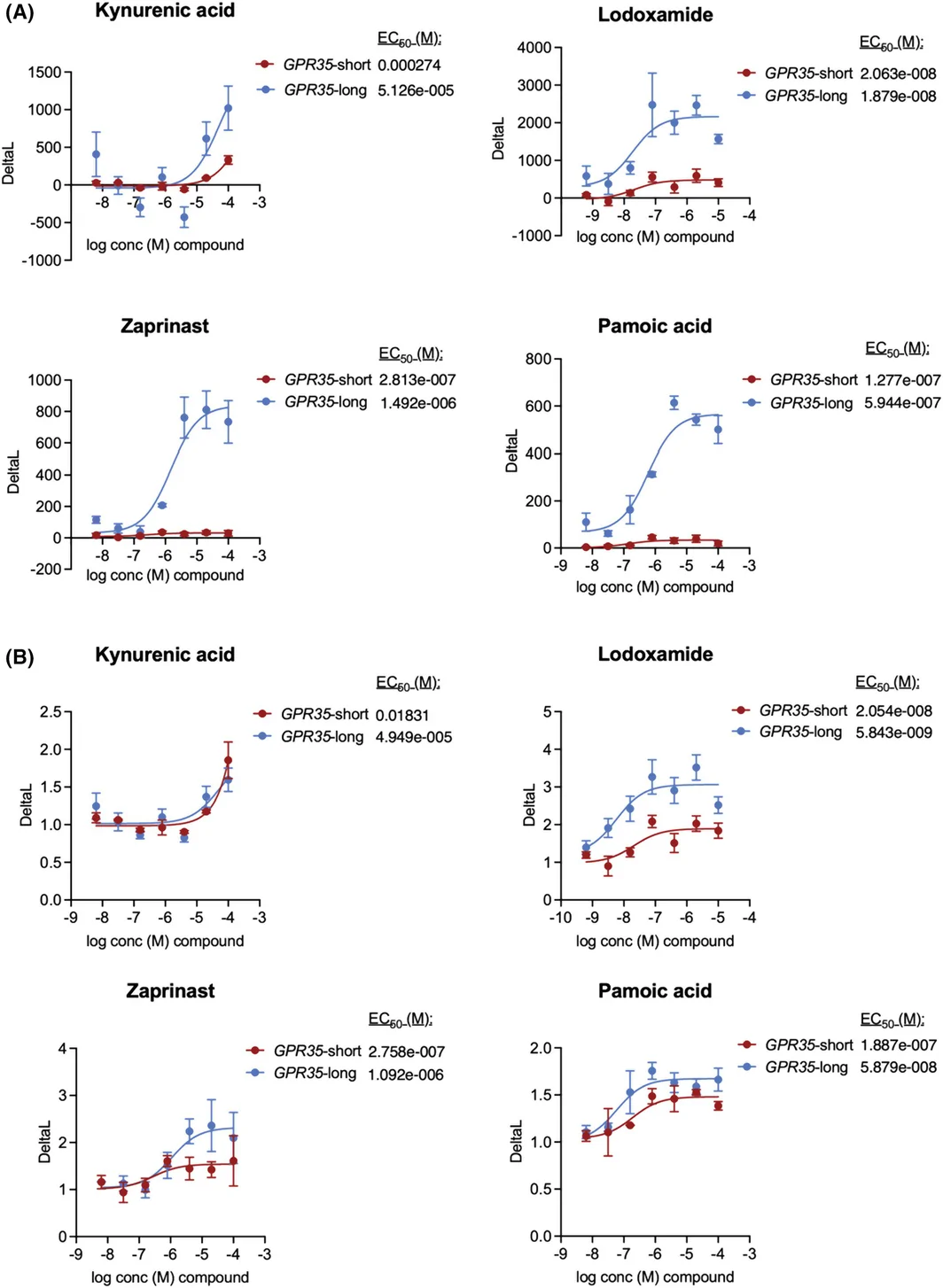
GPR35‐long exhibits increased constitutive, and ligand‐induced arrestin recruitment compared with reference isoform. Ligand‐dependent GPR35 signaling activity was assessed using the NanoBit luciferase assay by Promega. HEK‐293 cells were co transfected with either GPR35‐short or GPR35‐long C‐terminally fused to SmBit and beta‐arrestin N‐terminally fused to LgBit. Arrestin recruitment by either GPR35 isoform was then determined by measuring luciferase activity (luminescence) and addition of GPR35 ligands kynurenic acid, zaprinast, pamoic acid and lodoxamide. *N* = 3 independent experiments for all panels, each performed in duplicate wells. All data represented as mean ± s.e.m. Dose–response curves were generated using the graphpad software and a standard slope sigmoidal log(agonist) vs. response equation using baseline corrected absolute luminescence values (A) or response relative to baseline (B).

**Fig. 8:**
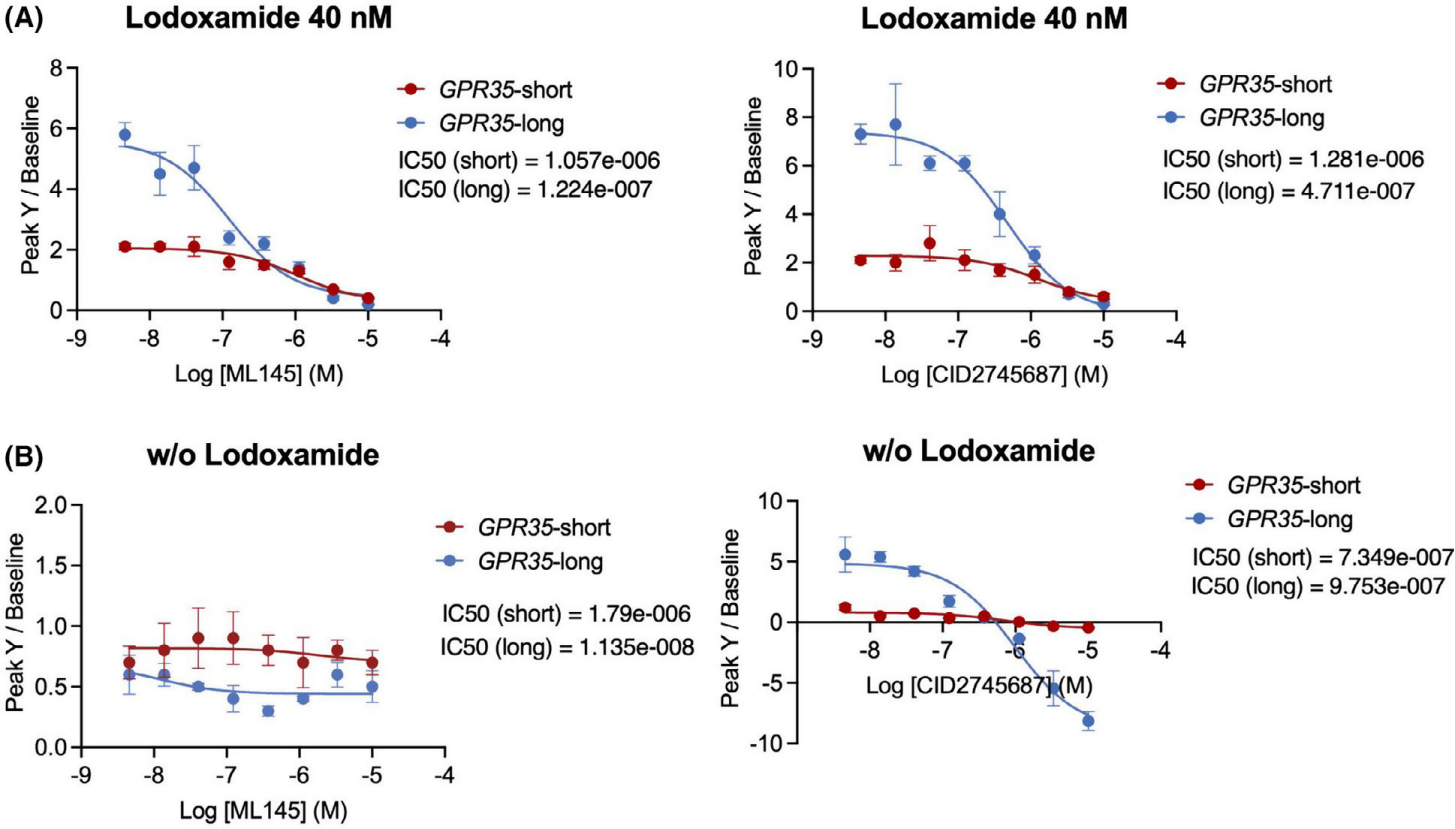
Increased constitutive activity of isoform can be effectively blunted by CID‐2745687 high‐affinity inverse agonist. Specific antagonists of GPR35 signaling activity were assessed using the NanoBit luciferase assay by co transfecting GPR35‐short or GPR35‐long C‐terminally fused to SmBit and beta‐arrestin N‐terminally fused to LgBit. (A) Inhibition of arrestin recruitment by either GPR35 isoform was then determined by measuring luciferase activity (luminescence) and simultaneous addition of 40 nm GPR35 lodoxamide and GPR35 antagonists ML‐145 and CID‐2745687. (B) Inhibition of constitutive arrestin recruitment by either GPR35 isoform was then determined by measuring luciferase activity (luminescence) and addition of GPR35 antagonists ML‐145 and CID‐2745687 alone. *N* = 3 independent experiments for all panels, each performed in duplicate wells. All data represented as mean ± s.e.m. Dose–response curves were generated using the graphpad software and a standard slope sigmoidal log(agonist) vs. response equation using baseline corrected absolute luminescence values.

### CID‐2745687 selectively suppresses *GPR35* ‐long‐associated signaling and metabolic phenotypes

Having demonstrated that *GPR35*‐long exhibits heightened constitutive β‐arrestin recruitment and is associated with increased cellular metabolic activity and proliferation, we next asked whether these phenotypes could be pharmacologically suppressed using the inverse agonist CID‐2745687.

We verified the previously obtained data via modifying the β‐arrestin assay and changing the constructs by expressing *GPR35*‐short and *GPR35*‐long as C‐terminal LgBit fusion proteins and using N‐terminal SmBit arrestin 2 constructs to rule out any artifacts caused by potentially variable expression levels of fusion proteins. Furthermore, we used the high affinity agonist zaprinast when examining CID‐2745687 blocking efficacy and again observed significantly increased constitutive and ligand‐induced β‐arrestin recruitment by GPR35‐long compared with GPR35‐short (Fig. 9A). Treatment with CID‐2745687 in the presence of zaprinast showed a significant reduction of ligand‐induced β‐arrestin recruitment by GPR35‐long compared with GPR35‐short‐expressing cells (Fig. 9A,B). In the absence of agonist, CID‐2745687 also significantly reduced constitutive signaling by GPR35‐long compared with GPR35‐short‐expressing cells (Fig. 9C). We then phenotypically investigated blocking constitutive GPR35 signaling in HEK‐293 cells overexpressing both TGFP‐tagged *GPR35* isoforms individually by using the Seahorse Mito Stress test and basal metabolic rate as a functional readout. We found that treatment with CID‐2745687 significantly reduced the metabolic rate in cells overexpressing *GPR35*‐long, whereas CID‐2745687 had no effect on cells overexpressing *GPR35*‐short or the *GPR35* negative parental cell line (Fig. 9D). We then evaluated the inhibitory potential of CID‐2745687 in Caco‐2 cells. CID‐2745687 treatment led to a significant reduction in basal metabolic rate in *GPR35* WT Caco‐2 cells and had no significant effect on *GPR35* knock out cells (Fig. 9E). Additionally, we detected a significant reduction of proliferation of CID‐2745687‐treated GPR35 WT cells that was absent in *GPR35* knock out cells (Fig. 9F).

**Fig. 9:**
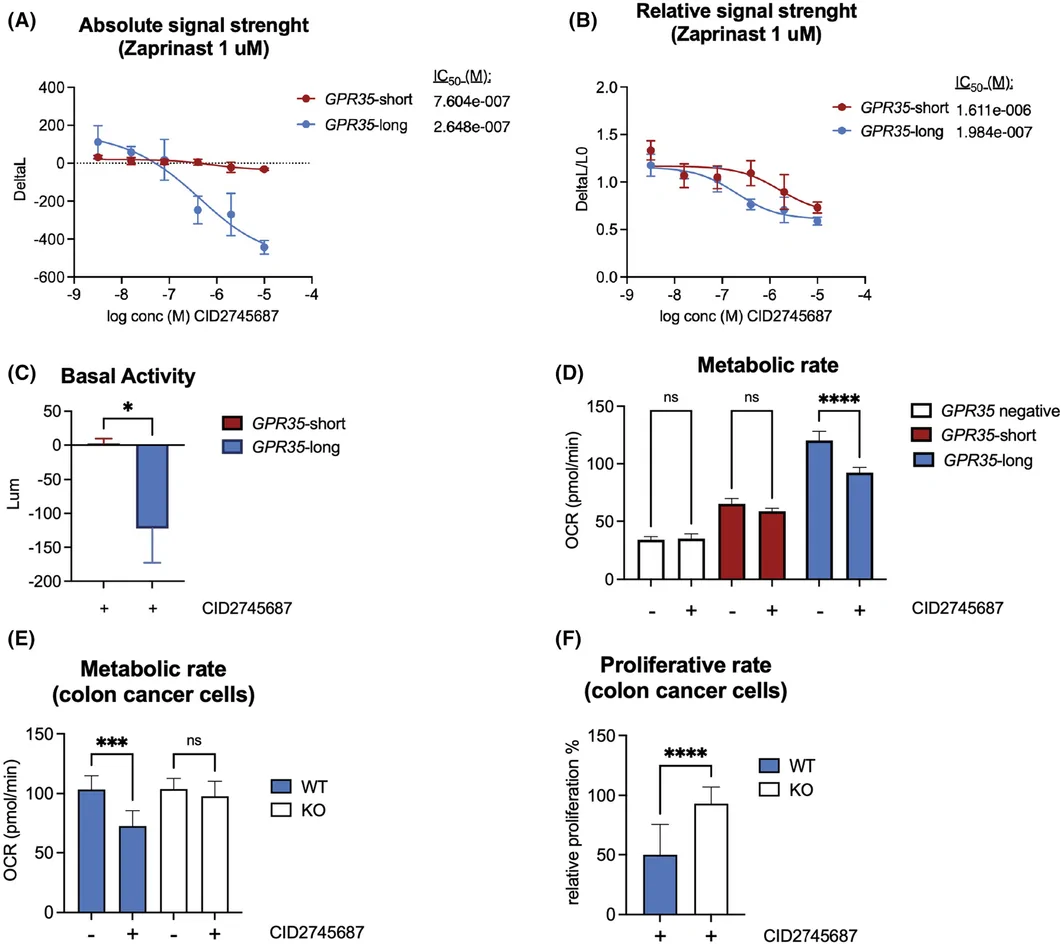
Small molecule inverse agonist CID‐2745687 dampens *GPR35*‐long‐associated metabolic and proliferative phenotypes in colorectal cancer cells. Inverse antagonism of CID2745687 was assessed using the NanoBit luciferase assay by co transfecting GPR35‐short or GPR35‐long C‐terminally fused to LgBit and beta‐arrestin N‐terminally fused to SmBit. Inhibition of arrestin recruitment by CID‐2745687 on either GPR35 isoform was then determined by measuring luciferase activity (luminescence) and simultaneous addition of 1 μm GPR35 zaprinast and CID‐2745687. Dose–response curves were generated using the graphpad software and a standard slope sigmoidal log(agonist) vs. response equation using baseline corrected absolute luminescence values (A) or response relative to baseline (B) and by comparing constitutive luminescence levels after addition of 10 μm CID‐2745687 (C). *N* = 3 independent experiments for panels A, B and C, each performed in duplicate wells. (D) The metabolic impact of CID‐2745687 on GPR35‐short‐ or GPR35‐long‐expressing cells was analyzed using real‐time metabolic profiling by Seahorse technology. Cellular respiration measured as oxygen consumption rate (OCR) using the Seahorse Mito stress test during sequential treatment with oligomycin, FCCP and antimycin/rotenone in GPR35‐expressing HEK‐293 cell clones compared with GPR35‐negative parental cells. Bar chart indicates basal respiration (metabolic rate). *N* = 3 biological replicates (clones) performed in triplicate wells. (E) The metabolic impact of CID‐2745687 on colorectal cancer cell line Caco‐2 expressing GPR35‐long was determined by Seahorse technology and compared with GPR35 knockout Caco‐2 cells. Bar chart indicates basal respiration (metabolic rate) comparing GPR35 wild‐type and GPR35 knockout Caco‐2 cells after addition of 10 μm CID‐2745687. *N* = 3 biological replicates (clones) performed in triplicate wells. (F) Proliferation of wild‐type and GPR35 knockout Caco‐2 cells was measured by standard 3‐(4,5‐dimethylthiazol‐2‐yl)‐2,5‐diphenyltetrazolium bromide (MTT) assay. Bar chart indicates relative proliferation of 10 μm CID‐2745687 treated wild‐type compared with GPR35 knockout cells. *N* = 3 biological replicates (clones) performed in triplicate wells. All data represented as mean ± s.e.m. Statistical significance was calculated by two‐tailed Student's *t*‐test using individual data, *P < 0.05, **P < 0.01, ***P < 0.001, ****P < 0.0001.

## Discussion

The present study characterizes GPR35‐long as a functionally distinct receptor isoform with preferential epithelial expression and provides mechanistic and proof‐of‐concept evidence linking it to tumor‐associated transcriptional, metabolic, and signaling in gastrointestinal tissues. These findings do not establish *GPR35*‐long as an *in vivo* tumor driver or a validated therapeutic target but define a set of isoform‐specific cellular phenotypes and identify a pharmacological tool capable of modulating them.

A central question motivating this study was whether the cancer‐associated upregulation of *GPR35* observed in prior reports [4, 6, 16] could be attributed to a specific isoform. Our data indicate that *GPR35*‐long is predominantly expressed in epithelial cells, particularly those of the gastrointestinal and biliary tracts, whereas *GPR35*‐short is the predominant isoform in immune cells. This differential tropism had been hypothesized previously [6, 16] but had not been extensively examined experimentally. Using transcript‐specific quantitative expression analyses, isoform‐specific peptide detection by mass spectrometry, and immunohistochemistry with a custom antibody validated in overexpression systems, we determined this isoform distribution across cell lines, primary cells, and tissue samples. The preferential expression of *GPR35*‐long in epithelial cells of colorectal and CCA lines, together with its elevated levels in colon mucosa biopsies, is consistent with the documented roles of epithelial *GPR35* in barrier function, proliferation, and metabolism [22, 33], and suggests that *GPR35*‐long may be the primary isoform through which these functions are mediated in epithelial tissues. Importantly, elevated *GPR35*‐long expression in tumor compared with normal epithelium, observed here by immunohistochemistry in a small clinical cohort, is consistent with prior reports linking *GPR35* expression to a worse prognosis in CRC [6]. These tissue‐based findings should be considered supportive rather than definitive given the sample size. Given the known challenges of GPCR antibody validation in native tissues [34], the antibody‐based data should be considered within the broader multi‐layered validation framework described above, recognizing that overexpression‐based validation establishes construct selectivity but does not fully confirm endogenous specificity.

To investigate the functional consequences of isoform‐specific *GPR35* expression, we performed transcriptomic profiling as a discovery‐oriented approach to identify pathway‐level signatures that could explain and predict the cellular phenotypes observed in downstream functional assays. Overexpression of *GPR35*‐long or *GPR35*‐short individually in the *GPR35*‐negative HEK‐293 background allowed controlled isoform comparison in an engineered, reductionist system, and the resulting transcriptional profiles differed markedly between isoforms, extending approaches used to characterize isoform‐specific signaling properties in heterologous systems [35, 36, 37]. *GPR35*‐long expression was associated with enrichment of pathways related to monocarboxylic acid transport, including pyruvate shuttling into and within mitochondria, small molecule catabolism, and reactive oxygen species metabolism, all of which are consistent with upregulated oxidative phosphorylation. In contrast, *GPR35*‐short expression enriched pathways related to locomotion and chemotaxis, consistent with its predominant immune‐cell expression and the established role of *GPR35* in modulating neutrophil and dendritic cell migration [1, 10]. Direct isoform comparison additionally identified enrichment of gene sets related to epithelial–mesenchymal transition (EMT) and ECM organization in *GPR35*‐long‐expressing cells, including wound healing, fibrosis, and angiogenesis‐associated signatures. These processes are relevant to tumor progression and stromal interaction in CRC [38]. PDGF signaling, which was also enriched in *GPR35*‐long‐expressing cells, has been associated with mesenchymal‐like CRC phenotypes [39]. These transcriptional findings should be interpreted as reflecting intrinsic isoform‐specific signaling capacity in an engineered system rather than direct evidence of endogenous tumor‐cell mechanisms, and pathway‐level associations require experimental follow‐up to establish mechanistic links.

The transcriptional profile of *GPR35*‐long predicted a metabolic phenotype that was directly confirmed by Seahorse extracellular flux analysis. *GPR35*‐long‐expressing HEK‐293 cells displayed significantly increased mitochondrial and glycolytic ATP production compared with both *GPR35*‐short‐expressing cells and the parental cell line, with elevated maximal respiration, spare respiratory capacity, and glycolytic reserve. Previous work from our group and others demonstrated metabolic roles for *GPR35* in ischemic protection and glycolysis, but without isoform resolution [2, 22, 40, 41]. The present data suggest that these metabolic effects are specifically associated with the *GPR35*‐long isoform, and that the transcriptomic pathway enrichments identified above reflect underlying functional differences between isoforms rather than incidental findings. The increased proliferation observed in *GPR35*‐long‐expressing HEK‐293 cells in MTT assays is consistent with this enhanced bioenergetic capacity, and with the enrichment of wound healing and proliferation‐associated gene sets in the transcriptomic data, although the precise downstream mechanisms linking *GPR35*‐long signaling to these phenotypes remain to be defined.

To assess whether these phenotypes were relevant in a disease‐appropriate epithelial context, we examined transcriptional and metabolic consequences of *GPR35* loss in Caco‐2 CRC cells using CRISPR/Cas9‐mediated KO. *GPR35* deletion resulted in substantial transcriptional changes, with upregulation of ECM organization pathways and high‐level induction of *TGFB2*. This is consistent with the EMT‐related signatures observed in *GPR35*‐long‐overexpressing HEK‐293 cells, and with the association of GPR35‐long expression with the mesenchymal CRC consensus molecular subtype 4, which is defined by TGF‐β production and stromal invasion [38]. These convergent findings across gain‐ and loss‐of‐function systems strengthen the notion that *GPR35*‐long is associated with transcriptional programs relevant to mesenchymal‐like CRC biology. Notably, oxygen consumption rates were not markedly different between GPR35‐KO and WT Caco‐2 cells despite the upregulation of metabolic pathway genes in KO cells. This may reflect metabolic compensation, as reprogrammed and flexible metabolism is a well‐established feature of CRC [42], and loss of one metabolic input may be buffered by transcriptional upregulation of alternative pathways. It should be noted that while these KO data demonstrate *GPR35* dependence, they do not distinguish between isoform‐specific contributions; isoform‐selective re‐expression experiments in the KO background would be required to definitively attribute the endogenous phenotypes to *GPR35*‐long. However, the HEK‐293 overexpression experiments performed in a *GPR35*‐null background with short and long isoforms introduced separately, provide an isoform‐resolved gain‐of‐function comparison where the convergence of pathway signatures across both systems supports the overall conclusion.

GPCR isoforms arising from alternative splicing are increasingly recognized as functionally distinct entities capable of diversifying signaling outcomes in response to the same ligand [35, 43, 44, 45, 46]. N‐terminal extensions in particular have been shown to influence receptor conformation and transducer coupling bias, as demonstrated for *CXCR3* isoforms, where the long isoform with an N‐terminal extension shows markedly distinct beta‐arrestin recruitment compared with the short form [36, 37]. The present study extends this framework to *GPR35*. Having established isoform‐associated transcriptional and metabolic phenotypes, we investigated the signaling properties of *GPR35*‐long as a potential mechanistic basis for these observations. Using a NanoBiT β‐arrestin recruitment assay in HEK‐293 cells, a well‐established reductionist platform for GPCR signaling studies, we found that *GPR35*‐long exhibits increased constitutive and ligand‐induced β‐arrestin 2 recruitment compared with *GPR35*‐short, across multiple high‐affinity agonists. This enhanced receptor‐arrestin coupling was observed at baseline in the absence of ligand, suggesting that the additional N‐terminal 31 amino acids of GPR35‐long confer a conformational preference for active‐state β‐arrestin coupling. Whether this heightened constitutive β‐arrestin activity mechanistically drives the transcriptional and metabolic phenotypes described above remains to be established. β‐arrestin signaling can activate ERK1/2 and other downstream effectors that regulate transcriptional programs and cellular metabolism, providing a plausible mechanistic hypothesis [47, 48, 49, 50, 51], but the precise intermediaries linking GPR35‐long β‐arrestin recruitment to the pathway enrichments and bioenergetic changes observed here require future investigation. Taken together, the signaling data demonstrate that GPR35‐long is significantly more β‐arrestin biased than its short counterpart. This is in line with previous observations by colleagues [15]. In addition, our data suggest that its elevated constitutive activity may contribute to the tumor‐associated cellular phenotypes described in this study.

Given the increased constitutive signaling activity of GPR35‐long, we tested the ability of high‐affinity antagonists ML145 (CID‐2286812) [52] and CID‐2745687 [53] to suppress it. Both compounds effectively inhibited ligand‐induced β‐arrestin coupling, but only CID‐2745687 demonstrated significant inverse agonism, reducing constitutive GPR35‐long activity below basal levels. Inverse agonism has been reported for both compounds [54], but we could replicate this only for CID‐2745687, likely reflecting differences in the reporter system used. Critically, CID‐2745687 treatment selectively reduced the metabolic rate and proliferation in *GPR35*‐long‐overexpressing HEK‐293 cells but had no effect on *GPR35*‐short‐expressing cells or the parental line, directly linking the inverse agonist activity to the isoform‐specific metabolic phenotype. In Caco‐2 cells, CID‐2745687 reduced the basal metabolic rate and proliferation in WT but not *GPR35* KO cells, establishing GPR35 dependence of the pharmacological effect in a disease‐relevant epithelial model. These proof‐of‐concept data support the utility of CID‐2745687 as a pharmacological probe for *GPR35*‐long‐dependent biology and provide a rationale for further investigation of its potential in CRC models. Previous work has shown that CID‐2745687 attenuates anchorage‐independent growth in CRC cells via YAP/TAZ inhibition [55], and the present data suggest that suppression of *GPR35*‐long‐associated metabolic and proliferative activity may represent an additional mechanism of action warranting exploration.

The isoform‐specific framework established here also has implications for understanding *GPR35*‐associated inflammatory disease. Numerous *GPR35* single‐nucleotide polymorphisms have been associated with IBD, PSC, and ankylosing spondylitis [17, 20, 56], and the coding variant rs3749171 (T108M) was previously shown to increase *GPR35* activity in immune cells [22]. Given that this variant falls within the shared region of both isoforms, it will be important to determine whether its hyperactivating effect operates through *GPR35*‐short in immune cells, *GPR35*‐long in epithelial cells, or both. This is a distinction that could inform which cell types and tissues are most relevant to disease pathogenesis.

In summary, this work provides evidence that *GPR35*‐long is a functionally distinct, epithelial‐restricted receptor isoform associated with tumor‐related transcriptional, metabolic, and signaling phenotypes. The transcriptomic data served not merely as a descriptive exercise but as a mechanistic bridge: pathway enrichments in *GPR35*‐long‐expressing cells predicted and framed the bioenergetic phenotypes subsequently confirmed by functional metabolic assays, and convergent signatures across HEK‐293 overexpression and Caco‐2 KO systems strengthen the overall interpretation. Collectively, these findings provide mechanistic and proof‐of‐concept insights into isoform‐specific *GPR35* biology in the gastrointestinal epithelium, while highlighting the need for *in vivo* validation and isoform‐resolved rescue experiments to fully define the role of *GPR35*‐long in CRCC progression.

## Author contributions

JDR contributed to the investigation, methodology, visualization, analysis, data curation. MH contributed to the investigation and analysis. JK contributed to the investigation, visualization, and analysis. JEE contributed to the conceptualization, and review and editing. MS contributed to the methodology, investigation, and analysis. KH contributed to the analysis and data curation. XJ contributed to the conceptualization, review and editing, and supervision. CK contributed to the investigation. TAN contributed to the analysis, review and editing. AK and NCK contributed to the review and editing. JRH contributed to the conceptualization, review and editing, supervision, administration, and funding. THK contributed to the conceptualization, review and editing, supervision, administration, resources, and funding. FLJ contributed to the conceptualization, investigation, resources, review and editing. GS contributed to the conceptualization, methodology, investigation, visualization, analysis, resources, original draft, supervision, administration, and funding.

## Conflict of interest

T.H.K. has consulted for Falk Pharma (relationship ended). The remaining authors have no conflict to report.

## Supporting information


**Fig. S1.** Validation of a GPR35‐long‐specific custom antibody.
**Fig. S2.** *GPR35*‐long overexpression in HEK‐293 cells is associated with a distinct transcriptional signature compared with *GPR35*‐short.
**Fig. S3.** Transcriptional analysis of lodoxamide treatment in *GPR35* WT and KO Caco‐2 cells.

## Data Availability

The data supporting the findings of this study are available within the article and its Supporting Information files. Raw RNA sequencing files have been uploaded to the European Nucleotide Archive (ENA) under the project number PRJEB101778. Code for read processing and alignment, differential gene expression analysis, and gene set enrichment analysis has been uploaded to GitHub (https://github.com/jdronneberg/GPR35‐long‐transcriptomics.git).
