# Supplementary material for: An epithelial GPR35 isoform supports tumor‐associated transcriptional and metabolic phenotypes
Source: FEBS Lett. 2026 Aug 1;600(19):2876–98. doi: 10.1002/1873-3468.70423 (PMC13656332; doi:10.1002/1873-3468.70423)
Supplement: Supplementary file 1 [file FEB2-600-2876-s001.pdf]

Supplemental figures

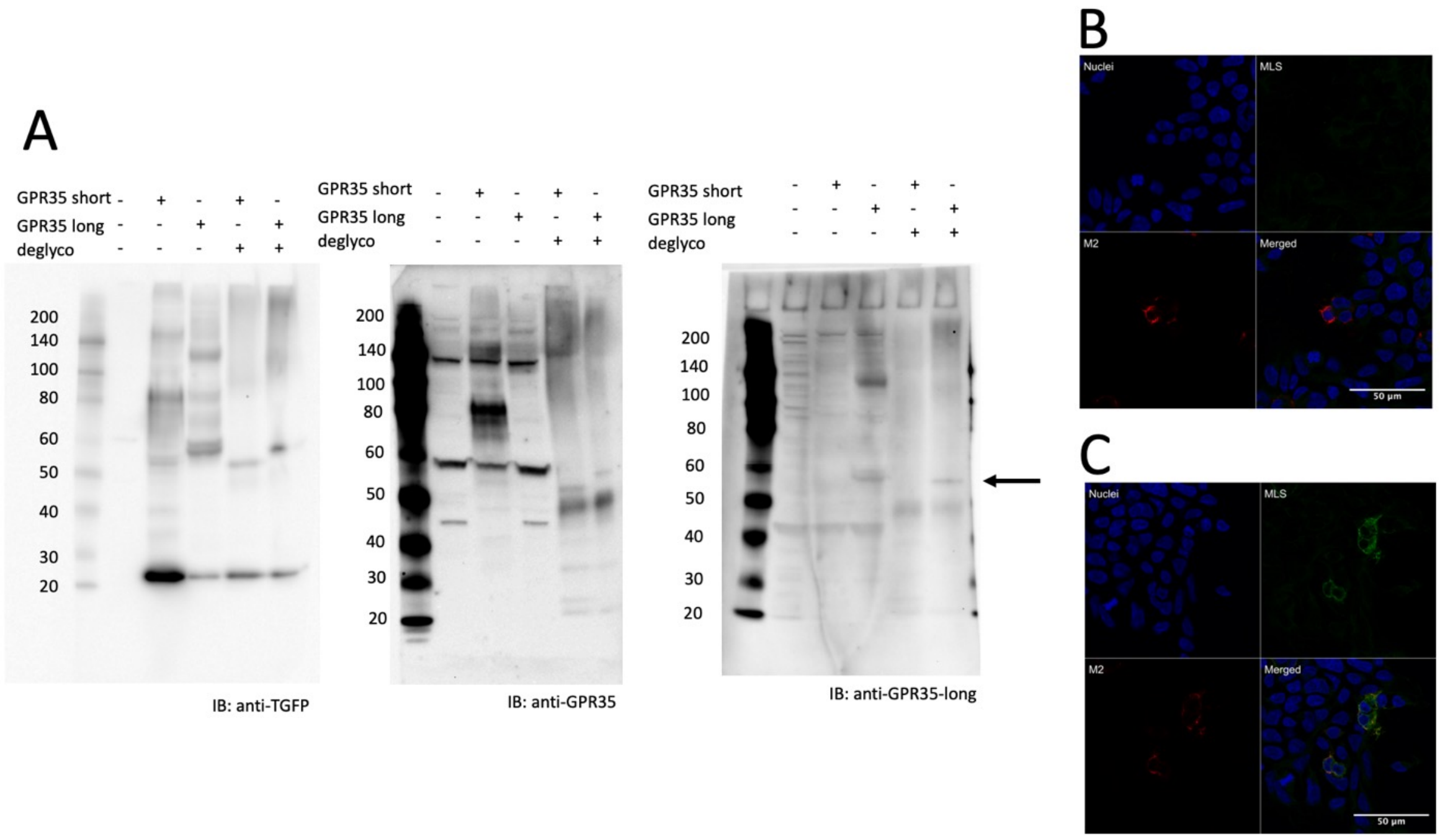

Supplementary Fig. S1. **Validation of a GPR35-long–specific custom antibody.** TGFP-tagged *GPR35*-short and *GPR35*-long were expressed in HEK-293 cells. **(A)** Whole-cell lysates were deglycosylated and analyzed by Western blot using anti-TGFP, anti-GPR35, and anti-GPR35-long antibodies in parallel. The arrow indicates the specific GPR35-long signal detected with the anti-GPR35-long antibody. **(B)** Methanol-fixed Flag-tagged *GPR35*-short–expressing cells were analyzed by immunofluorescence. Flag expression was visualized using the M2 antibody. The anti-GPR35-long antibody (MLS) did not stain *GPR35*-short–expressing cells. **(C)** Methanol-fixed Flag-tagged *GPR35*-long–expressing cells were analyzed by immunofluorescence. Flag expression was visualized using the M2 antibody. The anti-GPR35-long antibody (MLS) specifically stained *GPR35*-long–expressing cells. Scale bar (B, C): 50 µm.

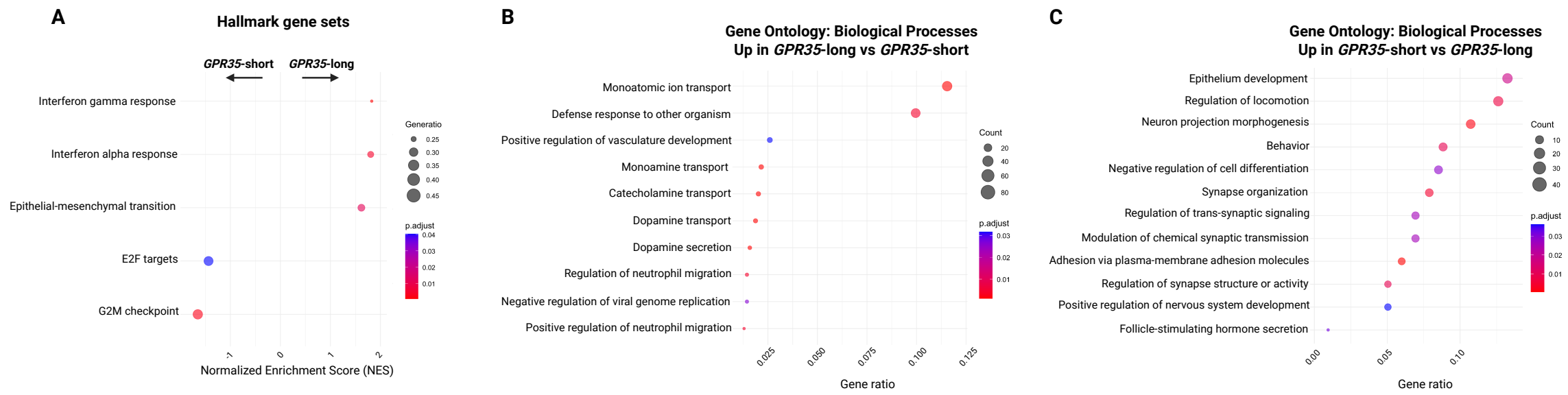

Supplementary Fig. S2. ***GPR35*-long overexpression in HEK-293 cells is associated with a distinct transcriptional signature compared to *GPR35*-short.** (A) Gene set enrichment analysis (GSEA) of significantly altered pathways in *GPR35*-long expressing cells versus *GPR35*-short. Enriched pathways are plotted based on normalized enrichment score (NES), with point size representing gene ratio and color indicating adjusted p-value. **(B)** Enrichment analysis of significantly altered pathways in *GPR35*-long expressing cells compared to *GPR35*-short expressing cells. **(C)** Enrichment analysis of pathways that are significantly downregulated in *GPR35*-long expressing HEK-293 cells compared to GPR35-short expressing HEK-293 cells. Enriched pathways are plotted based on gene ratio, with point size representing the number of downregulated genes found in the pathway and color indicating adjusted p-value.

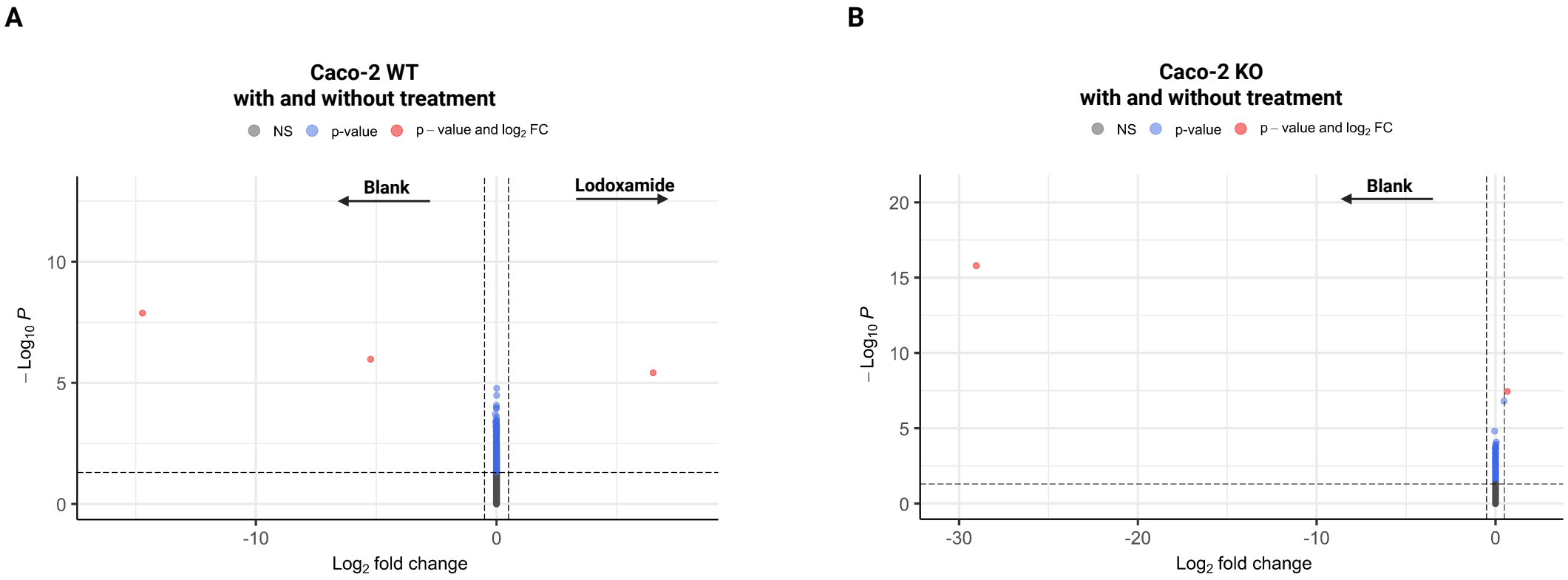

Supplementary Fig. S3. **Transcriptional analysis of Iodoxamide treatment in *GPR35* WT and KO Caco-2 cells.** Volcano plot displaying DEGs, following $\log_2$ fold change shrinkage, in *GPR35* WT (panel A), and in *GPR35* KO Caco-2 cells (panel B) with and without 1 μM Iodoxamide treatment. Genes with significant changes in expression (p-value < 0.01) and absolute $\log_2$ fold change > 0.5 are highlighted in red.
